# Genome-Wide Association Study and Selection Signatures Detect Genomic Regions Associated with Seed Yield and Oil Quality in Flax

**DOI:** 10.3390/ijms19082303

**Published:** 2018-08-06

**Authors:** Frank M. You, Jin Xiao, Pingchuan Li, Zhen Yao, Gaofeng Jia, Liqiang He, Santosh Kumar, Braulio Soto-Cerda, Scott D. Duguid, Helen M. Booker, Khalid Y. Rashid, Sylvie Cloutier

**Affiliations:** 1 Ottawa Research and Development Centre, Agriculture and Agri-Food Canada, Ottawa, ON K1A 0C6, Canada; xiaojin@njau.edu.cn (J.X.); lipingchuan@gmail.com (P.L.); gaofeng.jia@usask.ca (G.J.); liqiang.he@canada.ca (L.H.); 2 Morden Research and Development Centre, Agriculture and Agri-Food Canada, Morden, MB R6M 1Y5, Canada; zhen.yao@canada.ca (Z.Y.); scott.duguid@agr.gc.ca (S.D.D.); khalid.rashid@agr.gc.ca (K.Y.R.); 3 Department of Agronomy, Nanjing Agricultural University, Nanjing 210095, China; 4 Crop Development Centre, University of Saskatchewan, Saskatoon, SK S7N 5A8, Canada; helen.booker@usask.ca; 5 Brandon Research and Development Centre, Agriculture and Agri-Food Canada, Brandon, MB R7A 5Y3, Canada; Santosh.kumar@agr.gc.ca; 6 Department of Plant Science, University of Manitoba, Winnipeg, MB R3T 2N2, Canada; braulio.soto@cgna.cl; 7 Agriaquaculture Nutritional Genomic Center, CGNA, Temuco 4871158, Chile

**Keywords:** flax, genome-wide association study (GWAS), selective sweep, genotyping by sequencing (GBS), bi-parental population, single nucleotide polymorphism (SNP), seed yield, plant height, maturity, fatty acid composition

## Abstract

A genome-wide association study (GWAS) was performed on a set of 260 lines which belong to three different bi-parental flax mapping populations. These lines were sequenced to an averaged genome coverage of 19× using the Illumina Hi-Seq platform. Phenotypic data for 11 seed yield and oil quality traits were collected in eight year/location environments. A total of 17,288 single nucleotide polymorphisms were identified, which explained more than 80% of the phenotypic variation for days to maturity (DTM), iodine value (IOD), palmitic (PAL), stearic, linoleic (LIO) and linolenic (LIN) acid contents. Twenty-three unique genomic regions associated with 33 quantitative trait loci (QTL) for the studied traits were detected, thereby validating four genomic regions previously identified. The 33 QTL explained 48–73% of the phenotypic variation for oil content, IOD, PAL, LIO and LIN but only 8–14% for plant height, DTM and seed yield. A genome-wide selective sweep scan for selection signatures detected 114 genomic regions that accounted for 7.82% of the flax pseudomolecule and overlapped with the 11 GWAS-detected genomic regions associated with 18 QTL for 11 traits. The results demonstrate the utility of GWAS combined with selection signatures for dissection of the genetic structure of traits and for pinpointing genomic regions for breeding improvement.

## 1. Introduction

Flax (*Linum usitatissimum* L., 2*n* = 2*x* = 30) is a self-pollinating annual crop from the Linaceae family. It is a dual-purpose crop grown for its seed oil or stem fiber, resulting in two morphotypes: linseed and fiber. The linseed or flaxseed morphotype is rich in oil (40–50%) containing five main fatty acids: palmitic (PAL, C16:0, ~6%), stearic (STE, C18:0, ~2.5%), oleic (OLE, C18:1^∆9^, ~19%), linoleic (LIO, C18:2^∆9, 12^, ~13%), and linolenic (LIN, C18:3^∆9, 12, 15^, ~55%) [1,2]. Because of its high LIN content, linseed is the richest plant source of omega-3 fatty acid which is beneficial for reducing blood cholesterol levels and mitigating heart diseases in humans [3,4]. The same attributes make it ideal as industrial oil for use in paints, linoleum flooring, inks, soaps and varnishes [4].

Linseed breeding has focused on high seed yield (YLD), high oil content (OIL), and either high or low LIN content. Low LIN (2–4%) and high LIO (65–70%) lines have been developed through mutation breeding. NuLin™ 50 with 67.8% LIN (http://www.viterra.ca) and Omégalin with 65.8% (http://www.terredelin.com) are examples of high LIN linseed cultivars currently registered. Extremely low LIN lines such as Linola^TM^ or Solin^TM^ improve oxidative stability, making such cultivars suitable for the fabrication of margarine [3]. Since 1910, a total of 82 flax cultivars have been released in Canada [5]. These cultivars and elite breeding lines provide diverse genetic materials for dissecting the genetic architecture of oil biosynthesis and yield related traits in linseed. 

Several methods can be used to dissect the genetic architecture of crop traits. QTL or linkage mapping uses bi-parental populations to identify loci responsible for trait variation between parents based on a recombination-based genetic linkage map [6]. Bi-parental populations, such as F_2_, recombinant inbred line (RIL), doubled haploid (DH) and backcross (BC) populations, are the most widely used genetic resources for mapping QTL for traits of interest in self-fertilizing crops, including flax [7,8,9,10,11,12]. While bi-parental populations are easy to develop and have power for QTL detection, only the a limited number of alleles from the parental genotypes are analyzed in a single population, resulting in a narrow genetic base and low representation of allelic diversity [13]. In addition, genetic recombination is limited in these populations [14]. To increase the QTL dissection power, attempts have been made to expand the genetic diversity through other multiple-parent population types, such as nested association mapping (NAM) populations [15,16,17] and multi-parent advanced generation intercross (MAGIC) populations [18,19,20,21,22,23,24,25], while retaining the advantages of association mapping and bi-parental populations. However, the development of such populations requires careful planning and time. Natural populations that possess tremendous phenotypic diversity can be advantageous in genome-wide association study (GWAS) with various molecular markers in plants and animals [26,27,28,29,30,31]. Association mapping using a diverse germplasm panel overcomes the phenotypic diversity limitation of bi-parental populations, thereby increasing the QTL mapping power [32] but is impeded by low detection power of association of rare alleles. GWAS usually uses a natural population to investigate wider phenotypic variation for complex traits by taking advantage of ancient genetic recombination events in populations [33]. 

GWAS may be complemented by performing genome-wide selective sweep scan (GW3S) which identifies selection signatures that are beneficial for plant adaptation. A selective sweep is the reduction or elimination of variation among the nucleotides near a new beneficial mutation. Following strong positive natural selection or artificial selection during domestication or breeding, selective sweeps affect nearby linked alleles [34]. Ancient selective sweeps are relevant to natural evolution and domestication of crop species that are subjected to natural and artificial selective pressures and forced to adapt rapidly to new environments and thus drive speciation [35]. Breeding selects favorable alleles and retains them in new cultivars. These signatures of selection can be detected by a cross-population comparison approach [34]. Recent studies demonstrated that genomic regions that exhibit selection signatures are also enriched for genes associated with biologically important traits [36,37,38,39,40]. Thus, detection of selection signatures is emerging as an additional approach to identify and validate novel gene-trait associations [41].

Genetic regions associated with storage oil biosynthesis in flax have been studied based on QTL mapping using bi-parental populations. Several QTL responsible for oil content and fatty acid composition have been mapped in independent studies including the three populations used herein. The first population (BM) of 243 F_2:6_ recombinant inbred lines (RILs) from a cross between the Canadian linseed varieties CDC Bethune and Macbeth was used for a linkage mapping study and detected three QTL each for OLE and STE, two each for LIO and IOD, and one each for PAL, LIN and OIL with several QTL co-locating at the same locus [8]. The second population (EV) was a cross between E1747 and Viking. The third population (SU) was a cross between SP2047 (a yellow-seeded Solin^TM^ line with 2–4% LIN) and UGG5-5 (a brown-seeded flax line with 63–66% LIN) and comprised of 78 lines generated through DH method. It was used in a linkage mapping study using simple sequence repeat (SSR) markers which identified QTL for LIO, LIN and iodine value (IOD) co-locating on LG7 and LG16, and a QTL for PAL on LG9 [7]. The linkage-based studies from these populations provided numerous QTL for important traits but the QTL were generally far from the markers and poorly delimited because of the low resolution of the genetic maps [18,19,42].The three bi-parental populations were also used to construct a consensus genetic map [43], and to perform genomic selection [44] primarily using SSR markers. Because the three populations have been simultaneously phenotyped for several common agronomic and seed oil quality traits in the same environments (years/locations), we designed the present study to test the efficiency of the combined bi-parental population approach for GWAS and GW3S to detect genomic regions associated with seed yield and seed oil quality traits using genotyping by sequencing (GBS).

## 2. Results

### 2.1. Re-Sequencing and Genome-Wide SNPs

**Figure 1:**
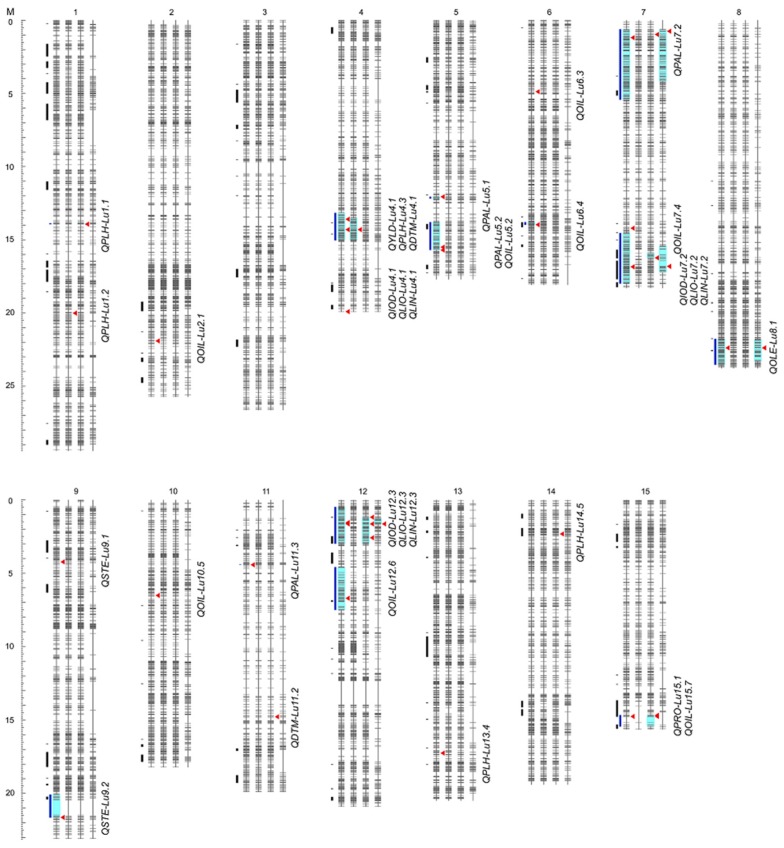
Distribution of 17,288 SNPs, 114 selective sweeps and 33 QTL on the 15 chromosomes of flax for each of three bi-parental populations BM, EV and SU and, for the merged population (BM + EV + SU). Four vertical bars from left to right for each chromosome represent the BM + EV + SU, BM, EV and SU populations, respectively. Short horizontal lines on bars represent SNPs. QTL regions are highlighted in cyan and by vertical blue lines. Red triangles identify QTL’s peak SNP. Selective sweeps are represented by short vertical black lines.

In the present study, a set of 260 genotypes (97 from the recombinant inbreeding line (RIL) population from a cross between CDC Bethune and Macbeth (BM), 91 from the RIL population from a cross between E1747 and Viking (EV) and 72 from the doubled haploid population from a cross between SP2047 and UGG5-5 (SU) along with the 5 of 6 parents except for the reference CDC Bethune) were re-sequenced using GBS to identify genome-wide single nucleotide polymorphism (SNP) markers on the chromosome-based flax pseudomolecules [45]. An average of ~57.7 million paired end reads were generated for each individual, corresponding to 5754 Mb sequences or 19.2× genome equivalents of the reference scaffolds (~302 Mb) [46] (Table S1). Paired-end reads of each genotype were aligned to the flax scaffolds [46], resulting in a total of 536,186 SNPs. After filtering off SNPs with minor allele frequency (MAF) <0.05 and genotyping rate <60% [47,48], 17,288 SNPs were retained on the flax pseudomolecules [45] (Table S2). Out of these, 15,284 segregated in BM, 15,397 in EV and 7568 in SU. The SNPs were mostly uniformly distributed across all 15 chromosomes (chr), ranging from 601 on chr11 to 1572 on chr13 (Figure 1, Table S2). Approximately 71.1% of all SNPs were located in intergenic regions, 16.2% were in introns and 12.7% were in exons (Table S2). These SNPs were used for further population structure analysis, GWAS and GW3S.

### 2.2. Whole-Genome Pattern of LD

**Figure 2:**
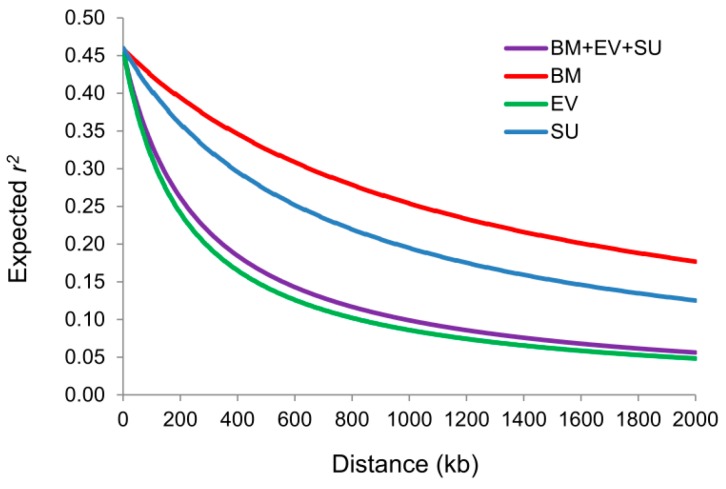
Intra-chromosome LD (*r*^2^) decay of SNP pairs over the entire flax genome as a function of physical distances (kb) of pair-wise SNPs for the three individual and merged populations. The curves are drawn based on a fitted non-linear model (see Section 4.2).

The LD and LD decay rates were analyzed for each population separately as well as the merged population using the filtered SNP data. The physical distances of pair-wise SNPs at which the LD *r*^2^ dropped to half were 1242, 223, 728 and 272 kb for BM, EV, SU and merged populations respectively. This indicated substantial variation in LD decay rate across populations (Figure 2). The average LD *r*^2^ of BM, EV, SU, and merged populations were 0.37, 0.26, 0.28 and 0.30, respectively, with the number of haplotype blocks for each population estimated at 599, 648, 206 and 1205, respectively (Table S3).

### 2.3. Genetic Diversity and Population Structure

Nucleotide diversity (*π*) was estimated at 41.52, 38.26 and 3.95 for the BM, EV and SU populations, respectively (Table 1), and was consistent with the number of SNPs identified from the three populations. A strong population-differentiation (*F_st_*) was observed at 0.44 between BM and SU and 0.48 between EV and SU. However, *F_st_* was weaker at 0.04 between the BM and EV (Table 1). 

**Figure 3:**
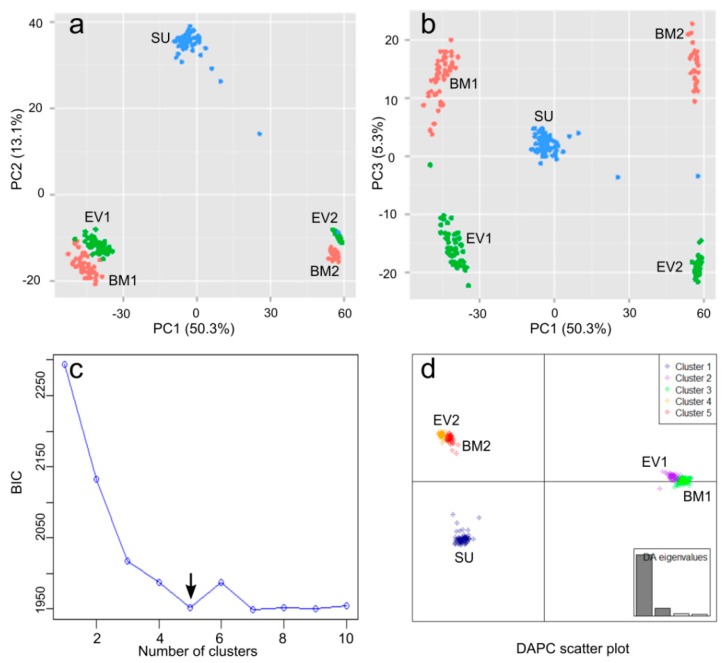
Principal component analysis (PCA) and discriminant analysis of principal components (DAPC) of the 260 individuals in three bi-parental populations (BM, EV and SU) based on 17,288 SNPs. (**a**) Bi-plot of the first and second principal components (PCs); (**b**) Bi-plot of the first and third PCs; (**c**) *k*-means clustering analysis based on 100 chosen PCs shows that the optimal number of clusters (*k*) is 5, that is where the Bayesian information criterion (BIC) is lowest (arrow); (**d**) DAPC scatter plot. Percentages in parentheses in the axis titles of (**a**) and (**b**) represent the variance explained by the respective PCs. Individuals from the BM and EV populations grouped into two subpopulations each, BM1 and BM2, and EV1 and EV2, respectively.

The genetic structure within the merged population was assessed based on the 17,288 SNP loci from the 260 individuals using two methods: principal component analysis (PCA) and discriminant analysis for principal components (DAPC). Bi-plots of the first three principal components of the PCA showed five distinct clusters (Figure 3a,b). The BM (BM1 and BM2) and EV (EV1 and EV2) populations each contained two sub-populations, while SU produced a single cluster. DAPC corroborated the same five clusters (Figure 3c,d). Therefore, a DAPC Q matrix based on the five clusters was generated and used as covariates to assess the population stratification in GWAS and phenotypic variation explained by the SNPs. 

### 2.4. $h_{SNP}^{2}$

Phenotypic variation of traits was largely explained by SNPs in the three individual and the merged populations (Table 2). The average $h_{SNP}^{2}$ for all 11 traits was 0.51. The largest $h_{SNP}^{2}$ values among the four populations ranged from 0.45 (YLD) to 0.90 (PAL). More than 80% of the phenotypic variation in one of the populations was explained by identified SNPs for days to maturity (DTM), IOD, PAL, STE, LIO and LIN. The $h_{SNP}^{2}$ varied from one population to another depending on the genetic variation between the two parents. For SU, little or no phenotypic variation was explained by SNPs for DTM, plant height (PLH) and STE. For EV, a relatively low phenotypic variation ($h_{SNP}^{2}$ < 0.1) was explained by SNPs for STE and OLE.

### 2.5. QTL Identified from 11 Traits

Using the best linear unbiased prediction (BLUP) values of phenotyping data collected from six to eight year/location environments with both generalized linear model (GLM) and mixed linear model (MLM), we identified a total of 33 QTL for 11 traits, one for YLD, eight for OIL, five for PLH, four for PAL, three each for IOD, LIO, and LIN, two each for DTM and STE, and one each for protein content (PRO) and OLE (Table 3, Figure 1, Figures S1 and S2). Thirty-one of the 33 QTL were detected using GLM and 13 with MLM (Tables S4 and S5). Of these latter 13, two QTL (QTL 18 for IOD and QTL 31 for LIN) were detected only by MLM, while the remaining 11 were identified by both MLM and GLM (Table S4).

Out of 33 QTL identified, 12, 6, 3 and 27 were from EV, SU, BM and merged population, respectively. Only six QTL were detected exclusively from two individual populations, including four (QTL 2 and 6 for PLH, QTL 8 for DTM and QTL 17 for OIL) from EV and two (QTL 3 and 4 for PLH) from BM. Eighteen were identified exclusively from the merged population. Ten QTL were detected simultaneously from the merged population and one or more individual populations (Tables S4 and S5).

QTL for YLD (QTL 1) was identified only in two environments (2010/Morden and 2012/Saskatoon) (Figure S2) but not in other environments or using BLUP estimates over the six year/location environments. We also performed GWAS for all other traits with phenotypic data from individual environments and obtained similar results with the QTL identified using BLUP values over multiple environments (Table S6).

### 2.6. QTL Effect Significance

**Figure 4:**
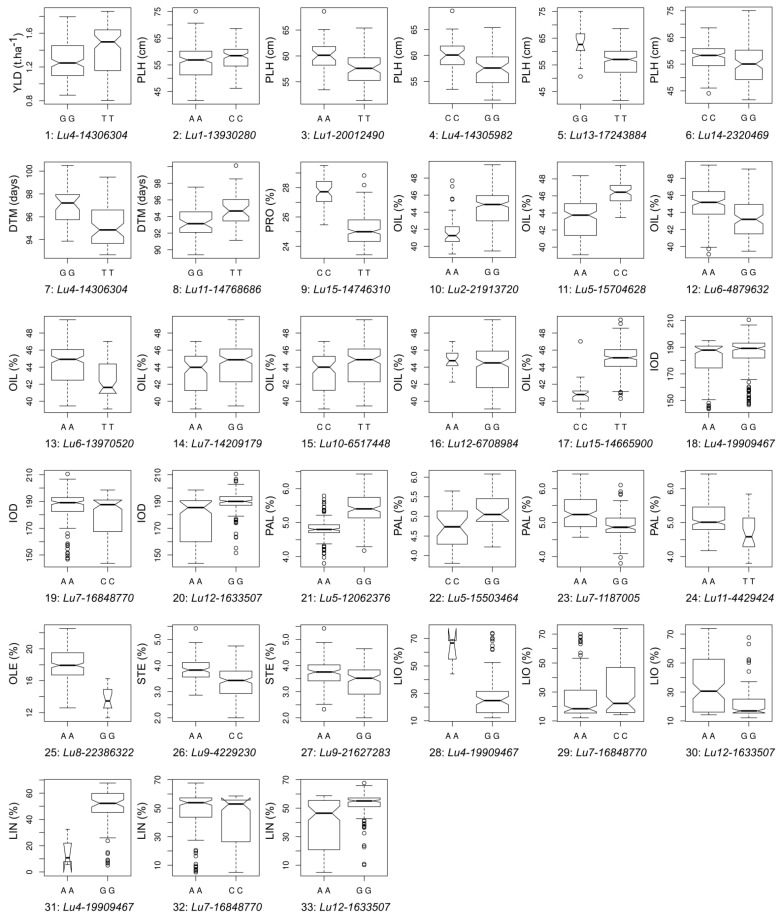
Trait performance of two contrasting haplotype pairs for each of 33 QTL identified from 11 traits. A QTL is represented by the peak SNP identified in the association study. The numbers of QTL correspond to QTL No in Table 3. The BLUP values of the 11 traits in the merged population were used except for PLH/QTL 3 and DTM/QTL 7 for which BM population was used, DTM/QTL 8 for which EV population was used, and PAL/QTL 22, LIO/QTL 28 and LIN/QTL 31 for which SU population was used. The box width is proportional to the size of the subpopulations. Phenotype differences between two contrasting haplotype pairs for each QTL are shown by boxes’ notches. For any given QTL, boxes’ notches that do not overlap indicate significant median differences at 95% confidence level.

**Figure 5:**
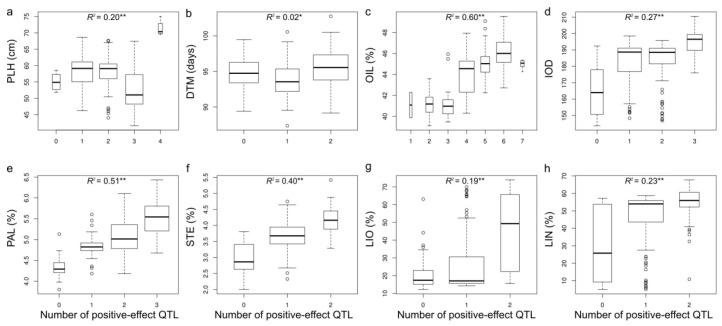
The relationship of phenotypes with the number of positive-effect QTL in individuals. Eight traits with two or more QTL identified were analyzed: (**a**) plant height (PLH), (**b**) days to maturity (DTM), (**c**) oil content (OIL), (**d**) iodine value (IOD), (**e**) palmitic acid content (PAL), (**f**) steric acid content (STE), (**g**) linoleic acid content (LIO), and (**h**) linolenic acid content (LIN). The BLUP values of the eight traits in the merged population were used. The correlation of phenotypes with the number of positive-effect QTL was calculated. * and ** represent statistical significance at 0.05 and 0.01 probability level.

To validate the QTL, we tested statistical significance of difference of phenotypes between two contrasting haplotype pairs for each QTL in the merged and individual populations and in different year/location environments. QTL effect differences between two contrasting haplotype pairs for all 33 QTL were significant (Figure 4, Table S7). We also assessed relationship of the number of pyramiding positive-effect QTL in individuals with trait phenotypes. Significant linear relations for all eight traits which had two or more QTL identified in this study were observed, showing primarily additive or accumulative QTL effects (Figure 5).

### 2.7. Pleiotropy of QTL

**Figure 6:**
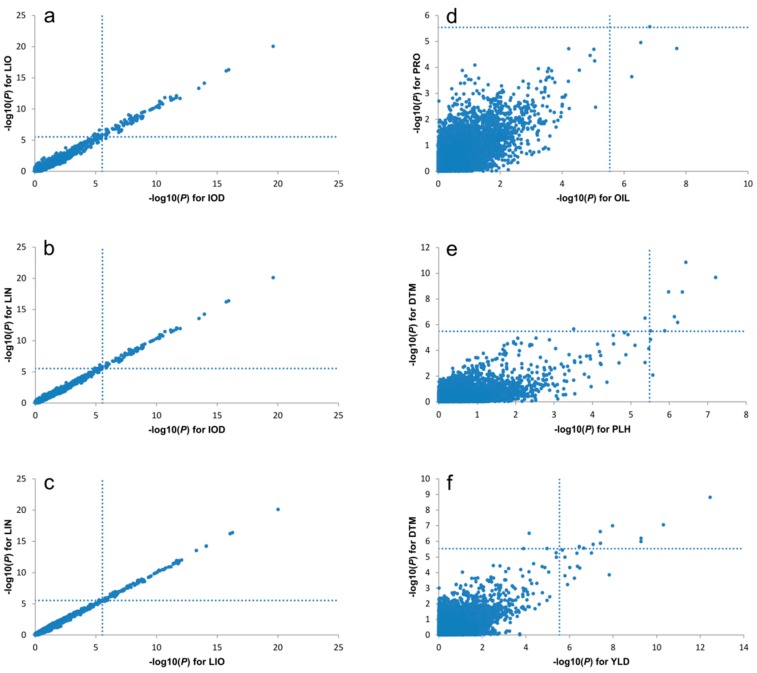
Relations of −log_10_(*P*) values of SNP markers between two traits showing pleiotropy or linkage relationship of SNP markers in different pairs of traits. (**a**) IOD vs. LIN; (**b**) IOD vs. LIO; (**c**) LIN vs. LIO; (**d**) OIL vs. PRO; (**e**) PLH vs. DTM; (**f**) DTM vs. YLD. Results of the GWAS using a GLM and data from the BM + EV + SU population for IOD, LIO, and LIN (**a**–**c**), the EV population for OIL and PRO (**d**), the BM population for PLH and DTM (**e**) and the BM + EV + SU population for DTM and YLD (**f**) are shown. The vertical and horizontal dashed lines show the cut-off value of significant SNP markers associated with a trait. YLD: seed yield (t ha^−1^); DTM: days to maturity; OIL: oil content (%); PRO: protein content (%); IOD: iodine value; LIO: linoleic acid content (%); LIN: linolenic acid content (%).

Sixteen of the 33 QTL co-located at six genomic regions concerning nine traits (Figure 1 and Figure 6, Table S8). QTL for PLH, DTH and YLD co-located on chr4. QTL for IOD, LIO and LIN co-located on chr4, 7 and 12. Chromosome 15 harbored QTL for OIL and PRO while chr5 had QTL for OIL and PAL.

### 2.8. Phenotypic Variation Explained by QTL

Phenotypic variations explained by individual QTL ($h_{QTL}^{2}$) were estimated (Table S4). Overall, the QTL explained 4 to 66% of the total phenotypic variation, with an average of 32.5% which is more than half of the average $h_{SNP}^{2}$ (51%). For five traits (IOD, LIO, LIN, PAL and OIL), QTL explained an average of 61% of the variation (Table 2 and Table S4). We also estimated the phenotypic variation explained by all QTL for a trait ($h_{GWAS}^{2}$) (Table 2). In the merged population, the QTL explained 48–73% of the phenotypic variation for OIL, IOD, PAL, LIO and LIN but only 8–14% for PLH, DTM and YLD.

### 2.9. Candidate Genes Underlying QTL

Based on the GWAS results, we investigated the genes annotated in the flax genome [54] in an attempt to predict candidate genes from loci significantly associated with each trait. The genomic locations of SNP markers at the peaks of the QTL were scanned within a 500 Kb window in either direction to constitute a subset of genes from which we deduced a candidate gene list based on *a priori* knowledge of their function(s). Candidate genes were identified for every QTL except for the YLD QTL (Table 3). We discovered seven candidate genes underlying QTL for DTM on chr4. The QTL for PLH harbors five candidate genes of completely different function. The genes underlying QTL for fatty acid composition include *KCS14-2*, *FAD3a*, and *FAD3b* for IOD/LIN/LIO, *KCS12-3* and *KAS Ic-1* for PAL, *KCS9-1* and *KCS1-1* for OLE, and *KCS18-2* and *SAD1* for STE.

### 2.10. Selection Signatures in Bi-Parental Populations

**Figure 7:**
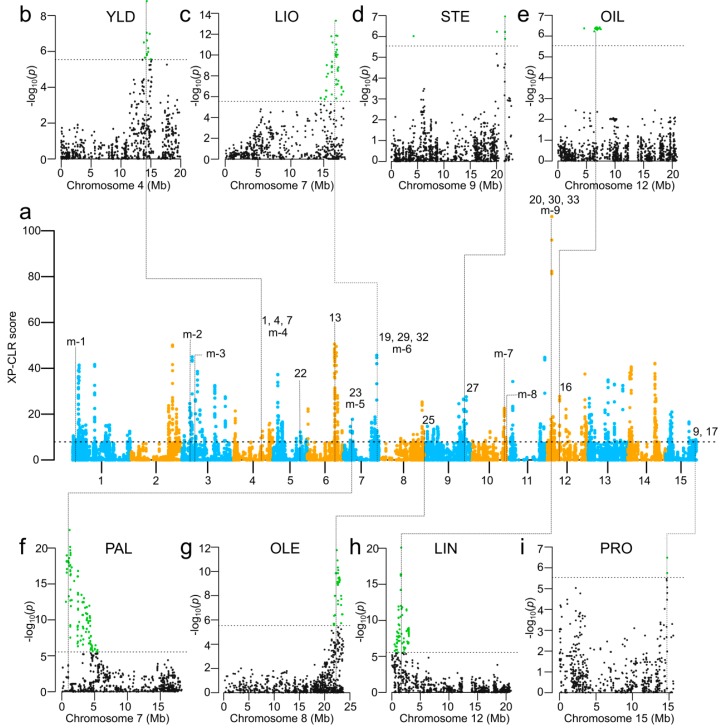
Genome-wide selective sweep scan using XP-CLR between BM and EV (**a**), and Manhattan plots of QTL overlapping with selective sweeps for (**b**) seed yield (YLD), (**c**) linoleic acid content (LIO), (**d**) steric acid content (STE), (**e**) oil content (OIL), (**f**) palmitic acid content (PAL), (**g**) oleic acid content (OLE), (**h**) linolenic acid content (LIN), and (**i**) protein content (PRO). QTL associated with selective sweeps are also labeled on peaks of selective sweeps. The numbers represent the QTL numbers listed in Table 3. Multiple numbers on the same peak represent genomic regions co-located with more than one trait. The labels ‘m-#’ represent the genomic regions associated with QTL previously identified and listed in Table S11. The green dots on Manhattan plots represent significant SNPs.

A GW3S was performed to identify potential selection signatures during breeding improvement using XP-CLR [34]. Due to the high genetic diversity in BM and EV (Table 1) and large phenotypic differences between them (Table S9), GW3S between BM and EV was conducted. A total of 114 selection signatures with an average size of 226.3 kb were identified (Figure 1 and Figure 7, Table S10), accounting for 7.82% of the flax pseudomolecules (~316 Mb). These putative selection signatures overlapped with 11 GWAS-detected genomic regions associated with 18 QTL (Figure 1 and Figure 7).

Some selection signatures were also associated with previously identified QTL (Table S11). For example, the selection signatures were associated with 10 previously reported QTL (Figure 7). The signatures at position 2.45–2.46 Mb on chr1 overlapped with SNP marker *Lu1_2670961* linked to QTL *QSte.BM.crc-LG1* for STE; the ones at 4.74–4.77 Mb on chr3 overlapped with *Lu3_5950394*, a SNP linked to QTL *QOle.BM.crc-LG3-1*/*QLio.BM.crc-LG3* for OLE and LIO; signatures at 7.24–7.25 Mb on chr3 overlapped with SNP *Lu3_8415336* linked to QTL *QSte.BM.crc-LG3* for STE [8]; position 16.80–16.81 Mb on chr10 harbors signatures that overlap with SSR *Lu2262* linked to an unnamed QTL for OIL; finally, position 17.52–17.53 Mb on chr10 has selection signatures that coincide with SSR *Lu2746* linked to an unnamed QTL for LIN/IOD [53].

## 3. Discussion

### 3.1. QTL Associated with Seed Yield and Seed Oil Quality Traits

Thirty-three QTL were identified in the current study. Of which, nine QTL were identified in previous studies [7,8] for the same traits, including seed yield and seed oil quality traits. Cloutier et al. [7] detected six major QTL for LIO, LIN and IOD in SU population. These six QTL correspond to the two underlying genes, *FAD3a* and *FAD3b*. Some of these QTL were in close proximity on the same chromosome. We identified the same QTL by association mapping that were previously detected by linkage mapping [7] using the same phenotype and SNP genotype data in the SU population (Table 3). The refinement of flax pseudomolecule [45] between the linkage study and our current association study allowed reassignment of chr12 for LIO, LIN and IOD QTL which were previously assigned to LG16 [8]. In addition, the same QTL were also detected in the EV population as well as the merged population. Our association study also validated three QTL for YLD, DTM and PAL which were previously identified using linkage mapping using SSRs and SNPs [8,9] and from the association mapping using a flax core collection population with SSR markers [53] (Table 3). These verified QTL for fatty acid composition, seed yield and maturity demonstrate the feasibility of the association mapping method to detect QTL in a bi-parental population as well as a multi-parent population.

An additional 24 novel QTL were detected in our current study which were not discovered in previous studies using individual BM or SU populations. These new QTL were detected using the merged population which greatly increased the population size, thereby enhancing the association power and resolution for QTL detection. We noted that only two QTL were discovered from the BM population alone. This is likely the result of significantly reduced representation of lines re-sequenced from BM population [8]. The discovery of new QTL demonstrates that GWAS using multiple bi-parental populations is equally or more efficient for QTL detection than QTL mapping using single bi-parental populations alone.

We tested the statistical significance of QTL effects for all 33 QTL identified for the 11 traits and found that all effect differences were significant. We also observed significant positive correlation between the number of positive-effect QTL and corresponding trait phenotypes in individuals for eight traits from which had two or more QTL were identified (Figure 4 and Figure 5, Table S7). These results not only corroborate the significance of the QTL but also demonstrate that effects of QTL in an individual performed additively, suggesting that marker-assisted selection (MAS) for these QTL would be effective in breeding. Thus, we listed the flanking sequences of these QTL in Table S12 for MAS purpose.

### 3.2. Pleotropic QTL Associated with Seed Yield and Quality Traits

Six genomic regions associated with more than one trait were identified. QTL for IOD, LIO, and LIN were concurrent on chromosomes 4, 7 and 12; QTL for YLD, PLH, and DTM co-located on chr4; QTL for PRO and OIL were on chr15 and QTL for PAL and OIL were on chr5 (Figure 1 and Figure 6, Table S8).

IOD is a measure of the degree of unsaturation of the oil that is calculated from the GC-derived fatty acid composition. Thus, breeding lines with high LIN normally show high IOD [7] due to the high correlation between IOD, LIO, and LIN [44] (Table S13). QTL co-located at the same genomic regions indicate that the traits may be controlled by the same gene or tightly linked genes. The two genomic regions on chromosomes 7 and 12 harbor the two fatty acid desaturase genes, *FAD3a* and *FAD3b*. These genes are responsible for linoleic and linolenic acid composition [52,55].

PLH and DTM are complex traits that considerably impact the adaptability, biomass, and economic yield of agricultural crops [56,57]. In soybean, one QTL that strongly associated with both PLH and DTM traits was identified with an SNP at 45.0 Mb position on chromosome 19 and it harbors the candidate gene *DT1*, which is homolog to *Arabidopsis terminal flower 1* (*TFL-1*, AT5G03840) [56]. Based on in silico gene annotation, the *DT1* homolog are located on chromosomes 6 and 8 in flax but no QTL for either PLH or DTM were identified on these two chromosomes. This could be due to the lack of functional polymorphism(s) at those loci among the parents of our three populations. However, a different genomic region on chr4 harbours five candidate genes for PLH and seven for DTM, raising the possibility that PLH and DTM are controlled by tightly linked genes in flax. The same genomic region was also associated with YLD. Because plant height and maturity affect seed yield, it is not surprising that QTL for PLH, DTM and YLD were mapped to the same locus. This pleiotropic relationship between YLD and DTM was previously validated [8] (Table 3).

Inheritance of seed oil content is complicated due to its quantitative nature. The seed oil content was directly affected by fatty acid composition traits, such as PAL, STE, OLE, LIO, and LIN, or indirectly by several major agronomic traits, such as seed yield and protein content [58]. Significant correlations of OIL were observed with PAL (−0.57; *p* = 0) and PRO (−0.70; *p* = 0) (Table S13). OIL is also usually negatively correlated with PRO in oilseed crops [59]. Of the eight QTL associated with oil content, two co-located with QTL for PAL on chr5 and for PRO on chr15, respectively.

### 3.3. Phenotypic Variation Explained by SNPs and QTL

SNP heritability ($h_{SNP}^{2}$) for a trait is the total proportion of phenotypic variance explained by additive contributions from genome-wide SNPs. A method for estimating $h_{SNP}^{2}$ for a complex trait was initially proposed in 2011 [60,61] and implemented in GCTA (Genome-wide Complex Trait Analysis) software [61]. Since then, the method has been applied to many quantitative traits largely in human and animal genetic studies [62,63]. The method was also used to estimate phenotypic variance explained by a subset of SNPs selected by *p*-values from GWAS in an independent sample [64]. However the estimate of variance explained by the SNP subsets ascertained by the *p*-values from GWAS in the same sample may be inflated due to positive correlation between true SNP effects and estimation errors (personal communication to the GCTA author, Jian Yang). However, as the GCTA-based heritability estimation method includes the population structure effect in the linear model and also considers heritability estimates to be irrelevant to the number of SNPs used [60,61], the accuracy of estimates should be higher than those obtained simply using the simple multivariate regression adopted in most GWAS of plant traits. In the current study, for the first time we applied this method to estimate $h_{SNP}^{2}$ for 11 agronomic and seed quality traits in three bi-parental populations and a merged population. As the number of SNPs identified from a population depends on its genetic variation for the traits, the trait-associated $h_{SNP}^{2}$ estimates vary across populations and traits. Overall, seed yield had a lower $h_{SNP}^{2}$ than seed quality traits as expected considering the extent of genetic complexity of the former (Table 2). We also used the same method to estimate phenotypic variation explained by individual QTL ($h_{QTL}^{2}$) and by all QTL for a specific trait ($h_{GWAS}^{2}$). $h_{GWAS}^{2}$ measures the extent of the phenotypic variation explained by QTL compared to that of all SNPs. This comparison led to the conclusion that many QTL for PLH, DTM and YLD were not detected in our study but the QTL for seed quality traits identified herein likely represent major genetic regions or genes controlling these traits.

### 3.4. Selection Signatures Associated with Seed Yield and Seed Quality Traits

GW3S has been used for screening putative genomic regions under selection pressure caused by domestication or artificial selection [36,38]. Usually, contrasting genetic populations are compared (such as wild accessions vs. cultivated accessions, landraces vs. breeding lines) to identify the allele frequency differentiation between different populations. In this study, we alternatively used two contrasting bi-parental mapping populations and identified 114 selection signatures with an average size of 226.3 kb. Some of these selection signatures support nearly 50% of the 23 GWAS-detected genomic regions associated with 33 QTL. Some of the QTL identified by GWAS have no overlapping selection signatures, partially because the regions of QTL had XP-CLR (Cross Population Composite Likelihood Ratio) scores less than the predetermined cut-off values. On the other hand, many selection signatures have high XP-CLR scores but no associated QTL (Figure 7). These significant selection signatures may be associated with QTL for traits not included in this study. This is suggested by the fact that five previously identified genomic regions related to seven QTL overlapped with the selection signatures identified in our current study comparing BM and EV (Table S10). These putative selection signatures provide useful candidates for further QTL-trait association study. Our results combined with previous studies demonstrate that GW3S combined with GWAS is a powerful approach for dissecting genetic structure of breeding populations and for the identification of underlying genomic regions for breeding improvement. Using GWAS with bi-parental populations and selection signatures allowed the cross validation of QTL previously identified by other mapping methods and established the foundation for genomic assisted breeding in flax.

## 4. Materials and Methods

### 4.1. Plant Materials

Three bi-parental mapping populations of different genetic backgrounds served as genotype panel for the association study. The first population (BM) consisted of 243 F_6_-derived RILs generated by single seed descent from a cross between CDC Bethune and Macbeth. Its two parents are Canadian high-yielding conventional linseed cultivars with 55–57% LIN [65,66]. The second population (EV) contained 90 F_6_-derived RILs from a cross between E1747, an ethyl methanesulfonate (EMS)-induced low LIN breeding line [67], and Viking, a French fiber flax cultivar with ~55% LIN that was grown extensively in the 2000’s but deregistered in 2012. The third population (SU) is an F_1_-derived DH population of 78 lines obtained from a cross between the breeding line SP2047, from which a yellow-seeded Solin^TM^ 2047 with only 2–3% LIN has been derived, and breeding line UGG5-5, which is a high LIN line with 63–66% LIN [7,55]. BM was designed to study yield-related traits while EV and SU were intended for QTL identification for fatty acid composition and fiber traits.

### 4.2. Whole Genome Resequencing, SNP Calling, SNP Imputation and LD Analysis

Three populations consisting of 97 randomly selected lines from BM, 91 from EV, 72 from SU including five parents (one parent is the reference genome) were grown in growth cabinets with a 16-h light and 8-h dark cycle at 20/18 °C. DNA was extracted from young leaf tissue using the DNeasy 96 Plant kit (Qiagen, Mississauga, ON, Canada) according to the manufacturer’s instructions. The DNA was subsequently restricted, size-selected and quantified prior to the construction of the reduced representation libraries used for Illumina sequencing as previously described [47]. Reduced representation libraries from a total of 260 individuals of the three populations, i.e., 96 randomly selected from BM, 89 from EV, 70 from SU, and five parents (One parent CDC Bethune of BM is used as a reference genome) were re-sequenced by the Michael Smith Genome Sciences Centre of the BC Cancer Agency, Genome British Columbia (Vancouver, BC, Canada) using 100-bp paired-end reads on an Illumina HiSeq 2000 platform (Illumina Inc., San Diego, CA, USA).

SNP calling was performed using the revised AGSNP pipeline [47,48,68]. The flax scaffold sequences of cultivar CDC Bethune [46] were used as reference for read mapping. Then SNPs were called using SAMtools [69] and further filtered using a set of criteria such as mapped read depth, consensus base ratio, mapping quality score and homopolymers with a validation rate of 96.8% for the called SNPs as described in detail [47]. Finally SNPs with a MAF < 0.05 and a genotyping rate <60% were removed for further analysis. The coordinates of all SNPs were extracted from the chromosome-based flax pseudomolecules v2.0 [45]. Missing data for these filtered SNPs were imputed using Beagle v.4.2 [70].

Intra-chromosome LD (*r*^2^) was calculated using plink ver. 1.9 [71] with the parameters “-r2 -ld-window-kb 2000 -ld-window-r2 0”. Before LD calculation, SNP data were pruned using the parameter “--indep-pairwise 2000 50 0.9” to remove SNPs with high *r*^2^ (>0.9) in a 2000 kb window with step size of 50 SNPs. Pair-wise *r*^2^ values were plotted against the base pair distance, and curves of LD decay with distances of paired SNPs were fitted using a non-linear regression model [72] as follows:

(1) $$r^{2}=\;\frac{10+cd}{\left(2+\;cd\right)\left(11+cd\right)}\;\times \left\{1+\;\frac{\left(3+cd\right)\left(12+12cd+\;{\left(cd\right)}^{2}\right)}{n\left(2+cd\right)\left(11+cd\right)}\right\},$$

where *c* is the coefficient to be estimated, *d* is the distance between pair-wise SNPs, and *n* is the number of total gametes, corresponding to twice the number of individuals in a population. The R function *nls* was used to fit the model. The rate of LD decay for each population was determined from the fitted model at the half of the maximum LD (*r*^2^). Haplotype blocks were calculated using plink with the parameters “--blocks no-pheno-req --blocks-max-kb 2000”.

### 4.3. Differentiation and Stratification

Nucleotide diversity (*π*) of three bi-parental populations and genetic differentiation (*F_st_*) between the populations were estimated using the R package “PopGenome” [73]. The genetic structures of the three separate inbreeding populations and the combined population were assessed using both PCA and DAPC [74]. Analyses with DAPC included several steps: (1) PCA was conducted using the imputed SNPs. According to the curve of accumulative variances against the number of principle components (PCs), the optimum number of PCs was chosen at which the cumulative variance had no obvious increase; (2) *k*-means clustering analysis was performed based on the chosen PCs. To identify the optimal number of clusters, *k*-means was run sequentially with increasing *k* values and the Bayesian information criterion (BIC) was calculated for each *k*. The optimum *k* corresponded to the lowest BIC; (3) Discriminant analysis was conducted based on the chosen clusters and individuals were reassigned to the different clusters. The posterior probability of cluster membership was calculated based on the retained discrimination functions and used as the Q matrix for GWAS and heritability estimation. PCA was performed using the function implemented in TASSEL while DAPC was conducted using the R package “adegenet” 2.0 [75].

### 4.4. Phenotyping of Bi-Parental Populations

Individuals from the three populations were evaluated in field trials over four years (2009–2012) at two sites, Morden Research and Development Centre, Manitoba (MD) and Kernen Crop Research Farm near Saskatoon, Saskatchewan (SAS) in Canada. A type-2 modified augmented design (MAD) [76] was used for the field experiments from which phenotypic data were collected. The detailed experimental design was previously described [44,77]. All 243 individuals of the BM population were phenotyped in four years (2009–2012) and two sites (MD and SAS), while 86 individuals of the EV population and 72 individuals of the SU population were evaluated in three years (2010–2012) and two sites (MD and SAS).

Eleven common traits were evaluated in the three populations, including YLD, PLH, DTM, PRO, OIL, IOD and five fatty acid composition traits (OLE, PAL, STE, LIO, and LIN). PLH was measured from ground to the uppermost part of the plant at maturity. DTM was recorded from sowing to 95% of capsule maturity (seeds rattling in the capsules or bolls). Seed yield data were measured by harvesting two 0.5-m sections from rows located in the central part of each subplot (0.2 m^2^). A total of 1 g of seed from each line at each environment was sampled for OIL measurement and fatty acid composition. Methyl esters of fatty acids were prepared according to the American Oil Chemists’ Society (AOCS) Official Method Ce 2-66 [78] and fatty acid composition was measured by gas chromatography (GC) following AOCS Official Method Ce 1e-91. OIL was determined by nuclear magnetic resonance calibrated against the FOSFA extraction reference method. PRO was measured using near-infrared spectroscopy calibrated against the combustion analysis reference method and expressed on an N × 6.25 dry basis. Phenotyping of these seed quality traits has been previously described [53]. All phenotypic data from the field experiments and laboratory measurements were adjusted for soil heterogeneity as previously described based on the MAD pipeline [77]. The BLUP values over multiple environmental phenotypes estimated using R package “lme4” [79] were used for further association study analyses. The Shapiro-Wilk normality test was performed for all traits using the R function “shapiro.test”. All 11 traits followed approximately a normal or mixed normal distribution (Figure S3). Simple correlations among 11 traits were calculated using the function “rcorr” of the R package “Hmisc”.

### 4.5. Phenotypic Variation Explained by All SNPs

The phenotypic variation explained by all SNPs, denoted as $h_{SNP}^{2}$, was estimated for all traits based on the following mixed linear model [60] implemented in the GCTA software [61]:

(2) $$y=X\beta +g+\varepsilon \;\mathrm{with}\;\mathrm{its}\;\mathrm{variance}\;V=A\sigma_{g}^{2}+I\sigma_{\varepsilon }^{2}$$

where y is an *n* × 1 vector of phenotypes with *n* individuals in a population, X is the *n* × *n*.

structure matrix, ***β*** is a vector of fixed effects of population structure, including posterior probabilities of an individual assigning to a cluster in DAPC, g is an *n* × 1 vector of the total genetic effects of the individuals with g ~*N* (**0**, $A\sigma_{g}^{2}$), and ε is a vector of residual effects with ε ~*N* (**0**, $I\sigma_{\varepsilon }^{2}$). ***A*** is interpreted as the genetic relationship matrix (GRM) between individuals and estimated from SNPs. $\sigma_{g}^{2}$ is estimated using the restricted maximum likelihood (REML) method based on the GRM estimated from all SNPs. Thus, SNP heritability $h_{SNP}^{2}$ was estimated as

(3) $$h_{SNP}^{2}=\;\frac{\sigma_{g}^{2}}{\sigma_{g}^{2}+\sigma_{\varepsilon }^{2}}$$

### 4.6. Genome-Wide Association Study

GWAS was performed with the GLM and compressed MLM [80,81] implemented in TASSEL (v5.2) [82], which employs the EMMA and P3D algorithms to reduce computing time. For MLM, the kinship matrices for the merged population and the three single populations were calculated using TASSEL (v5.2) [82]. Manhattan plots and quantile-quantile (Q-Q) plots of GWAS were obtained using the R package “qqman” [83].

SNP markers for candidate QTL were determined based on the *p*-value for each marker estimated in the GLM or MLM analysis. The *p*-values were adjusted by the Bonferroni correction, being α (0.05)/No. of SNPs used in the analyses. Allele effects of significant markers were calculated as the difference between the average phenotypic values of homozygous alleles which were obtained directly from the TASSEL outputs. Candidate QTL were defined based on peaks of SNPs exceeded the significance threshold for the trait. The genomic region for a QTL was defined as a genome block spanning all significant SNPs.

The amount of phenotypic variation explained by significant QTL was estimated for all SNP markers within the QTL regions using the same method as described above [61], denoted as $h_{QTL}^{2}$. We similarly estimated phenotypic variation explained by all significant QTL for a single trait and denoted it $h_{GWAS}^{2}$.

### 4.7. Candidate Gene Mining

Genome-wide gene scan along chromosomes for significant QTL was performed to characterize the underlying genomic regions and identify candidate genes. First, all orthologous genes of the model species *Arabidopsis thaliana* were mapped to the flax genome using BLASTP of flax protein sequences against *A. thaliana* protein sequences at an E-value of 1.0 × 10^−10^. A total of 15,323 unique *A. thaliana* genes were mapped. A list of known flax or *A. thaliana* genes associated with our studied traits and their associations was drawn based on literature and database searches [49,51,84]. We investigated candidate genes within QTL regions or within a 500 kb window upstream and downstream of the peaks depending on the LD decay estimates. In addition, previously identified QTL (SSR markers) in flax [7,8,53] were mapped to the flax pseudomolecules to validate the QTL results from this study.

### 4.8. QTL Validation

Three approaches were applied to validate QTL identified by GWAS. The first approach was to compare our QTL with previously identified QTL as described above. The same QTL was inferred if two QTL were mapped to the same recombination block or haplotype block. The second approach tested the significance of difference of phenotypes between two contrasting haplotype pairs of a QTL in the populations. Statistically significant differences served to validate significant QTL. Both *t* and Wilcox non-parametric tests were performed using the R functions “t.test” and “wilcox.test” for each QTL in the merged and individual populations and in different year/location environments. To test the positive correlations of a trait upon pyramiding of QTL, a simple regression of the number of positive-effect QTL on phenotypic values of a trait was calculated. A positive-effect QTL in an individual meant that this individual possessed a positive effect allele for the QTL. The last approach was to perform genome-wide selective sweep scans to confirm QTL associated genomic regions as described below.

### 4.9. Genome-Wide Selective Sweep Scan

A WG3S was performed along chromosomes across two populations using the program XP-CLR [34]. Comparisons between BM and EV using XP-CLR were conducted. The genetic distances (cM) between SNPs were estimated using the integrated flax consensus genetic map [43], assuming uniform recombination between SNPs. For each chromosome, XP-CLR was executed with the parameters “-w1 0.005 100 100 1 -p1 0.7” to estimate XP-CLR scores for 100-bp windows. Each chromosome was then divided into 10-kb segments and the highest XP-CLR score from windows with at least one SNP were assigned to each 10-kb segment ($x_{max,i}$). If the XP-CLR scores ($x_{max,\;i}$ and $x_{max,\;i+1}$) of two adjacent 10-kb segments were greater than the 80th percentile ($x_{max,80th}$) of the genome-wide scores of all 10-kb fragments, then they were grouped as a single putative selective sweep. In addition, putative selective sweeps were also merged if they were separated by no more than one low score (<$x_{max,80th}$) segment. Merged selective sweeps were assigned the highest score from their merged 10-kb segments. These merged segments were further combined into a larger region if these segments belonged to the same peak in the genome-wide selective sweep plot (Figure 5a). Finally, the combined regions falling in the highest 10th percentile of all putative selective sweeps were considered differentially selected regions or selection signatures.

The selection signatures were compared to both our detected QTL and previously reported QTL on the genetic loci to find associations between them. Positions where the QTL corresponding markers were located were extended by 100 kb on both sides and then compared with the position of the selection signatures. The QTL and selection signatures were considered associated when they overlapped.

## Figures and Tables

**Table 1:** Genetic differentiation (*F_st_*) between three bi-parental (upper triangle elements) and nucleotide diversity (*π*) within these populations (diagonal elements).

Population	BM	EV	SU
BM	41.52	0.04	0.44
EV		38.26	0.48
SU			3.95

BM: CDC Bethune/Macbeth; EV: E1747/Viking; SU: SP2047/UGG5-5.

**Table 2:** Phenotypic variation explained by all SNPs ($h_{SNP}^{2}$) and identified QTL ($h_{GWAS}^{2}$) for 11 traits in different populations.

Trait	Population	$h_{SNP}^{2}\;\pm \;s$	No. QTL	$h_{GWAS}^{2}\;\pm \;s$
YLD	BM + EV + SU	0.43 ± 0.12	1	0.14 ± 0.09 ^§^
	BM	0.22 ± 0.25		
	EV	0.15 ± 0.24		
	SU	0.45 ± 0.21		
PLH	BM + EV + SU	0.53 ± 0.12	1	0.08 ± 0.11
	BM	0.76 ± 0.12	2	0.21 ± 0.15
	EV	0.76 ± 0.14	2	0.22 ± 0.18
	SU	0.06 ± 0.20		
DTM	BM + EV + SU	0.43 ± 0.13	1	0.10 ± 0.07
	BM	0.81 ± 0.11	1	0.18 ± 0.13
	EV	0.36 ± 0.24	1	0.18 ± 0.22
	SU	0.00 ± 0.20		
PRO	BM + EV + SU	0.51 ± 0.11	1	0.12 ± 0.16
	BM	0.52 ± 0.20		
	EV	0.34 ± 0.23	1	0.09 ± 0.12
	SU	0.58 ± 0.19		
OIL	BM + EV + SU	0.66 ± 0.09	7	0.62 ± 0.14
	BM	0.46 ± 0.22		
	EV	0.39 ± 0.21	1	0.08 ± 0.08
	SU	0.70 ± 0.15		
IOD	BM + EV + SU	0.80 ± 0.06	3	0.57 ± 0.10
	BM	0.49 ± 0.19		
	EV	0.78 ± 0.12	2	0.51 ± 0.14
	SU	0.66 ± 0.17	2	0.35 ± 0.18
PAL	BM + EV + SU	0.79 ± 0.06	4	0.48 ± 0.11
	BM	0.12 ± 0.26		
	EV	0.55 ± 0.20	1	0.09 ± 0.11
	SU	0.90 ± 0.07	1	0.56 ± 0.18
STE	BM + EV + SU	0.21 ± 0.15	2	0.41 ± 0.19
	BM	0.85 ± 0.09		
	EV	0.02 ± 0.14		
	SU	0.00 ± 0.22	1	
OLE	BM + EV + SU	0.55 ± 0.10	1	0.16 ± 0.13
	BM	0.36 ± 0.22		
	EV	0.09 ± 0.25		
	SU	0.72 ± 0.16	1	0.20 ± 0.19
LIO	BM + EV + SU	0.80 ± 0.06	3	0.73 ± 0.07
	BM	0.54 ± 0.20		
	EV	0.75 ± 0.13	2	0.54 ± 0.14
	SU	0.66 ± 0.17	2	0.36 ± 0.18
LIN	BM + EV + SU	0.80 ± 0.06	3	0.56 ± 0.09
	BM	0.49 ± 0.19		
	EV	0.76 ± 0.13	2	0.55 ± 0.14
	SU	0.66 ± 0.17	2	0.36 ± 0.18

YLD: seed yield; PLH: plant height; DTM: days to maturity; PRO: protein content; OIL: oil content; IOD: iodine value; PAL: palmitic acid content; STE: stearic acid content; OLE: oleic acid content; LIO: linoleic acid content; LIN: linolenic acid content; BM: CDC Bethune/Macbeth; EV: E1747/Viking; SU: SP2047/UGG5-5. ^§^ $h_{GWAS}^{2}$ of YLD was estimated based on the phenotypes in a single environment (Morden/2012). For all other traits, $h_{GWAS}^{2}$ was estimated based on the BLUP estimates of phenotypes.

**Table 3:** QTL and associated gene candidates.

Trait	QTL No.	QTL Name	Chr.	Start Position (bp)	End Position (bp)	XP-CLR Score	Known QTL or Marker	Candidate Gene IDs	Candidate Gene Location (bp)	Candidate Gene Name	Gene Annotation
YLD	1	*QYLD-Lu4.1*	4	13,594,936	14,968,389	12.54	*QYld.BM.crc-LG4* ^a^				
PLH	2	*QPLH-Lu1.1*	1	13,887,715	13,930,292						
	3	*QPLH-Lu1.2*	1	20,012,490	20,012,490			Lus10020835	19,610,837	*BRI1* [49]	Leucine-rich receptor-like protein kinase family protein
								Lus10020865	19,790,777	*GA2* [49]	Terpenoid cyclases/Protein prenyltransferases superfamily protein
	4	*QPLH-Lu4.3*	4	14,305,982	15,042,104	12.54		Lus10034358	14,006,288	*BIM2* [49]	BES1-interacting Myc-like protein 2
								Lus10041435	14,157,752	*MYB62* [49]	Myb domain protein 62
								Lus10041481	14,398,338	*LMCO4* [49]	Laccase/Diphenol oxidase family protein
								Lus10041794	15,920,170	*ROT3* [49]	Cytochrome P450 superfamily protein
								Lus10041801	15,948,434	*WAT1* [49]	Walls Are Thin 1
	5	*QPLH-Lu13.4*	13	17,243,884	17,243,884			Lus10030567	18,680,474	*GA2OX8* [49]	Gibberellin 2-oxidase 8
	6	*QPLH-Lu13.5*	14	2,320,469	2,320,469	40.61		Lus10021395	3,647,029	*HCT* [49]	Hydroxycinnamoyl-CoA shikimate/quinate hydroxycinnamoyl transferase
DTM	7	*QDTM-Lu4.1*	4	13,171,757	15,042,104	12.54	*QDm.BM.crc-LG4* ^a^	Lus10015766	13,094,864	*FLC* [50]	K-box region and MADS-box transcription factor family protein
								Lus10034461	13,434,121	*CDF3* [50]	Cycling DOF factor 3
								Lus10034370	13,933,421	*AP1* [50]	K-box region and MADS-box transcription factor family protein
								Lus10041483	14,411,103	*PFT1* [50]	Phytochrome and flowering time regulatory protein (PFT1)
								Lus10041500	14,512,085	*ATAN11* [50]	Transducin/WD40 repeat-like superfamily protein
								Lus10041540	14,716,950	*RGL1* [50]	RGA-like 1
								Lus10041595	14,966,739	*AP2* [50]	Integrase-type DNA-binding superfamily protein
	8	*QDTM-Lu11.2*	11	14,768,686	14,768,686						
PRO	9	*QPRO-Lu15.1*	15	14,746,288	14,746,310	8.50		Lus10030671	22,732,660	*WRI* [50]	Integrase-type DNA-binding superfamily protein
OIL	10	*QOIL-Lu2.1*	2	21,913,720	21,913,720						
	11	*QOIL-Lu5.2*	5	15,704,607	15,705,039						
	12	*QOIL-Lu6.3*	6	4,879,632	4,879,632						
	13	*QOIL-Lu6.4*	6	13,799,180	13,970,951	50.58					
	14	*QOIL-Lu7.4*	7	14,209,179	14,209,179						
	15	*QOIL-Lu10.5*	10	6,517,448	6,517,448						
	16	*QOIL-Lu12.6*	12	4,591,214	7,491,405	27.77					
	17	*QOIL-Lu15.7*	15	14,665,900	15,429,055	8.89		Lus10039906	19,833,852	*KCS14-2* [51]	3-ketoacyl-CoA synthase
IOD	18	*QIOD-Lu4.1*	4	19,909,467	19,909,467			Lus10039906	19,833,852	*KCS14-2* [51]	3-ketoacyl-CoA synthase
	19	*QIOD-Lu7.2*	7	15,346,458	17,977,459	45.70	*QIOD.crc-LG7* ^b^	Lus10038321	16,089,922	*FAD3a* [52]	Fatty acid desaturase
	20	*QIOD-Lu12.3*	12	489,561	2,981,642	106.22	*QIOD.crc-LG16* ^b^	Lus10036184	1,035,336	*FAD3b* [52]	Fatty acid desaturase
								Lus10023359	1,729,292	*FAH1* [50]	Fatty acid hydroxylase 1
PAL	21	*QPAL-Lu5.1*	5	12,062,376	12,182,441			Lus10029880	12,062,376	*KCS12-3* [51]	3-ketoacyl-CoA synthase
	22	*QPAL-Lu5.2*	5	13,797,851	15,668,995	12.14					
	23	*QPAL-Lu7.3*	7	624,461	5,423,691	17.74	*QPal.BM.crc-LG7* ^a^ *QPAL.crc-LG9* ^b^ *c79-s540_Lu2534* ^c^	Lus10001814	79,471	*KAS Ic-1* [51]	3-ketoacyl-acyl carrier protein synthase I
								Lus10028925	1,085,389	*KAS IIIb-2* [51]	3-ketoacyl-acyl carrier protein synthase III
								Lus10028885	1,262,079	*SUN1* [50]	SAD1/UNC-84 domain protein 1
	24	*QPAL-Lu11.4*	11	4,417,685	4,429,424			Lus10026345	4,333,672	*KCS7-1* [51]	3-ketoacyl-CoA synthase
OLE	25	*QOLE-Lu8.1*	8	21,782,841	23,527,563	12.64		Lus10006636	22,165,534	*KCS9-1* [51]	3-ketoacyl-CoA synthase
								Lus10006637	22,174,324	*KCS1-1* [51]	3-ketoacyl-CoA synthase
								Lus10018485	23,111,453	*DES-1-LIKE* [50]	Fatty acid desaturase family protein
STE	26	*QSTE-Lu9.1*	9	4,229,230	4,229,230			Lus10040333	4,275,842	*KCS18-2* [51]	3-ketoacyl-CoA synthase
	27	*QSTE-Lu9.2*	9	20,080,531	21,636,823	27.55		Lus10011877	20,059,127	*SAD1* [51]	Stearoyl acyl carrier protein desaturase
								Lus10011839	20,227,416	*FatA2-2* [51]	FatA acyl-ACP thioesterase
LIO	28	*QLIO-Lu4.1*	4	19,909,467	19,909,467			Lus10039906	19,833,852	*KCS14-2* [51]	3-ketoacyl-CoA synthase
	29	*QLIO-Lu7.2*	7	14,540,706	17,977,459	45.70	*QLIO.crc-LG7* ^b^ *c281-s185_ Lu566* ^c^	Lus10038321	16,089,922	*FAD3a* [52]	Fatty acid desaturase
	30	*QLIO-Lu12.3*	12	489,561	2,981,642	106.22	*QLIO.crc-LG16* ^b^ *Llio-LG12.3* ^c^	Lus10036184	1,035,336	*FAD3b* [52]	Fatty acid desaturase
LIN	31	*QLIN-Lu4.1*	4	19,909,467	19,909,467			Lus10039906	19,833,852	*KCS14-2* [51]	3-ketoacyl-CoA synthase
	32	*QLIN-Lu7.2*	7	14,540,719	17,977,459	45.70	*QLIN.crc-LG7* ^b^ *c281-s185_ Lu566* ^c^	Lus10038321	16,089,922	*FAD3a* [52]	Fatty acid desaturase
	33	*QLIN-Lu12.3*	12	489,561	2,981,642	106.22	*QLIN.crc-LG16* ^b^ *Llin-LG12.3* ^c^	Lus10036184	1,035,336	*FAD3b* [52]	Fatty acid desaturase
								Lus10023359	1,729,292	*FAH1* [50]	Fatty acid hydroxylase 1

^a^ QTL identified in [8]; ^b^ QTL identified in [7]; ^c^ QTL identified in [53]. All candidate genes are labelled by references.

## Supplementary Materials

Supplementary materials can be found at http://www.mdpi.com/1422-0067/19/8/2303/s1.

## Abbreviations

DH	doubled haploid
GBS	genotyping by sequencing
GW3S	genome-wide selective sweep scan
GWAS	genome-wide association study
IOD	iodine value
LD	linkage disequilibrium
LIN	linolenic acid
LIO	linoleic acid
MAF	minor allele frequency
MAGIC	multi-parent advanced generation intercross
NAM	nested association mapping
OIL	oil content
OLE	oleic acid
PAL	palmitic acid
QTL	quantitative trait loci
RIL	recombinant inbred line
SNP	single nucleotide polymorphism
SSR	simple sequence repeat
STE	stearic acid
YLD	seed yield
