# Supplementary material for: Genome-Wide Association Study and Selection Signatures Detect Genomic Regions Associated with Seed Yield and Oil Quality in Flax
Source: Int J Mol Sci. 2018 Aug 6;19(8):2303. doi: 10.3390/ijms19082303 (PMC6121305; doi:10.3390/ijms19082303)
Supplement: Supplementary file 1 [file ijms-19-02303-s001.zip › ijms-338884-supplementary-final check/Supplementary_figuresS1-S3-final check.docx]

**Genome-wide association study and selection signatures detect genomic regions associated
with seed yield and oil quality in flax**

**Frank M. You ^1,2,^*, Jin Xiao ^1,3^, Pingchuan Li ^1^, Zhen Yao ^2^, Gaofeng Jia ^1,4^, Liqiang He ^1^,
Santosh Kumar ^5^, Braulio Soto-Cerda ^6,7^, Scott D. Duguid ^2^, Helen M. Booker ^4^, Khalid Y. Rashid ^2^
and Sylvie Cloutier ^1,6,^***

^1^ Ottawa Research and Development Centre, Agriculture and Agri-Food Canada, Ottawa, ON K1A 0C6, Canada; [xiaojin@njau.edu.cn](mailto:xiaojin@njau.edu.cn) (J.X.); [lipingchuan@gmail.com](mailto:lipingchuan@gmail.com) (P.L.); [gaofeng.jia@usask.ca](mailto:gaofeng.jia@usask.ca) (G.J.); [liqiang.he@canada.ca](mailto:liqiang.he@canada.ca) (L.H.)

^2^ Morden Research and Development Centre, Agriculture and Agri-Food Canada, Morden, MB R6M 1Y5, Canada; [zhen.yao@canada.ca](mailto:zhen.yao@canada.ca) (Z.Y.); [scott.duguid@agr.gc.ca](mailto:scott.duguid@agr.gc.ca) (S.D.D.); [khalid.rashid@agr.gc.ca](mailto:khalid.rashid@agr.gc.ca) (K.Y.R.)

^3^ Department of Agronomy, Nanjing Agricultural University, Nanjing 210095, China

^4^ Crop Development Centre, University of Saskatchewan, Saskatoon, SK S7N 5A8, Canada; helen.booker@usask.ca

^5^ Brandon Research and Development Centre, Agriculture and Agri-Food Canada, Brandon, MB R7A 5Y3, Canada; [Santosh.kumar@agr.gc.ca](mailto:Santosh.kumar@agr.gc.ca)

^6^ Department of Plant Science, University of Manitoba, Winnipeg, MB R3T 2N2, Canada; [braulio.soto@cgna.cl](mailto:braulio.soto@cgna.cl)

^7^ Agriaquaculture Nutritional Genomic Center, CGNA, Temuco 4871158, Chile

***** Correspondence: [frank.you@agr.gc.ca](mailto:frank.you@agr.gc.ca) (F.M.Y.); [sylvie.cloutuer@agr.gc.ca](mailto:sylvie.cloutuer@agr.gc.ca) (S.C.);
Tel.: +1-613-759-1539 (F.M.Y.); +1-613-759-1744 (S.C.)


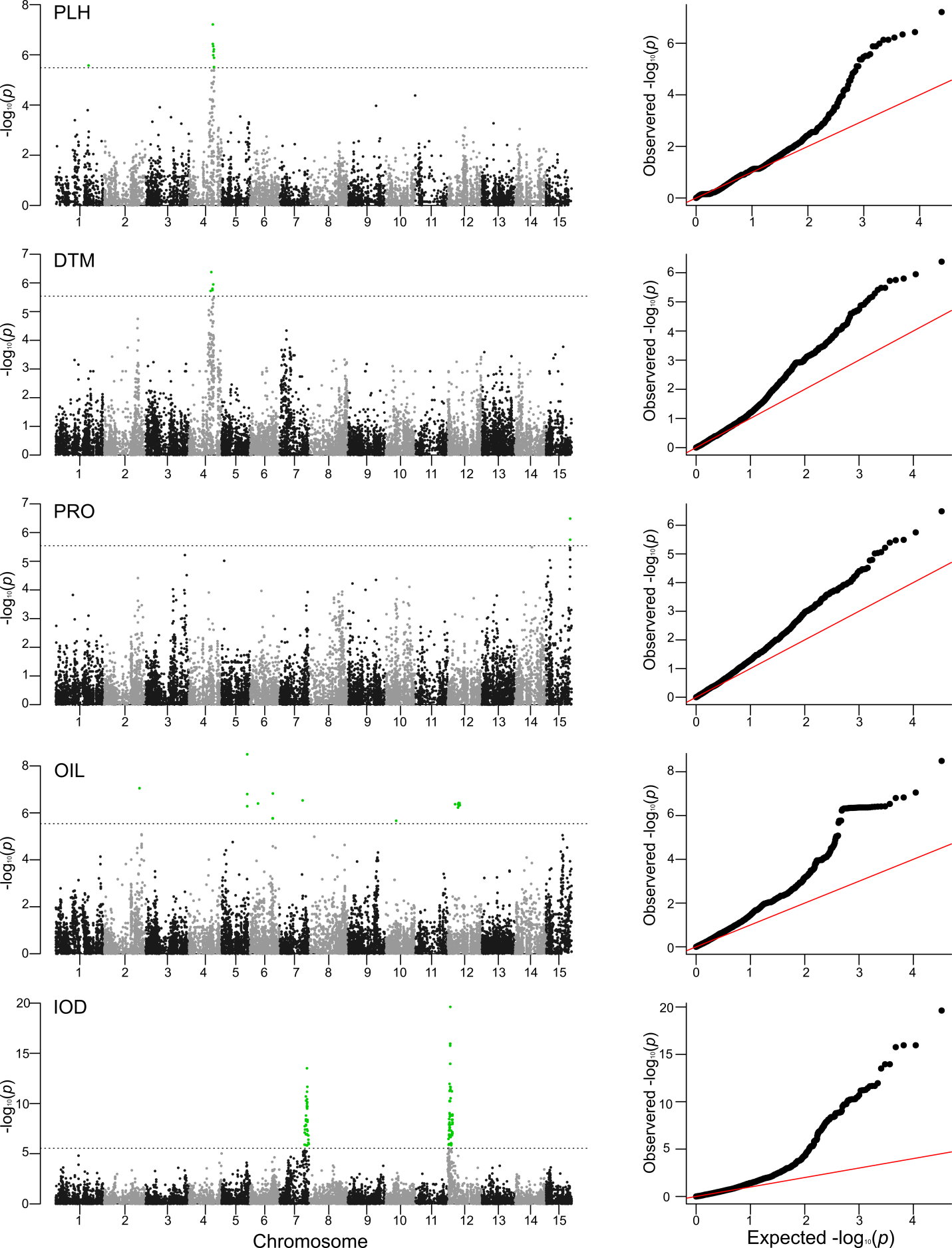


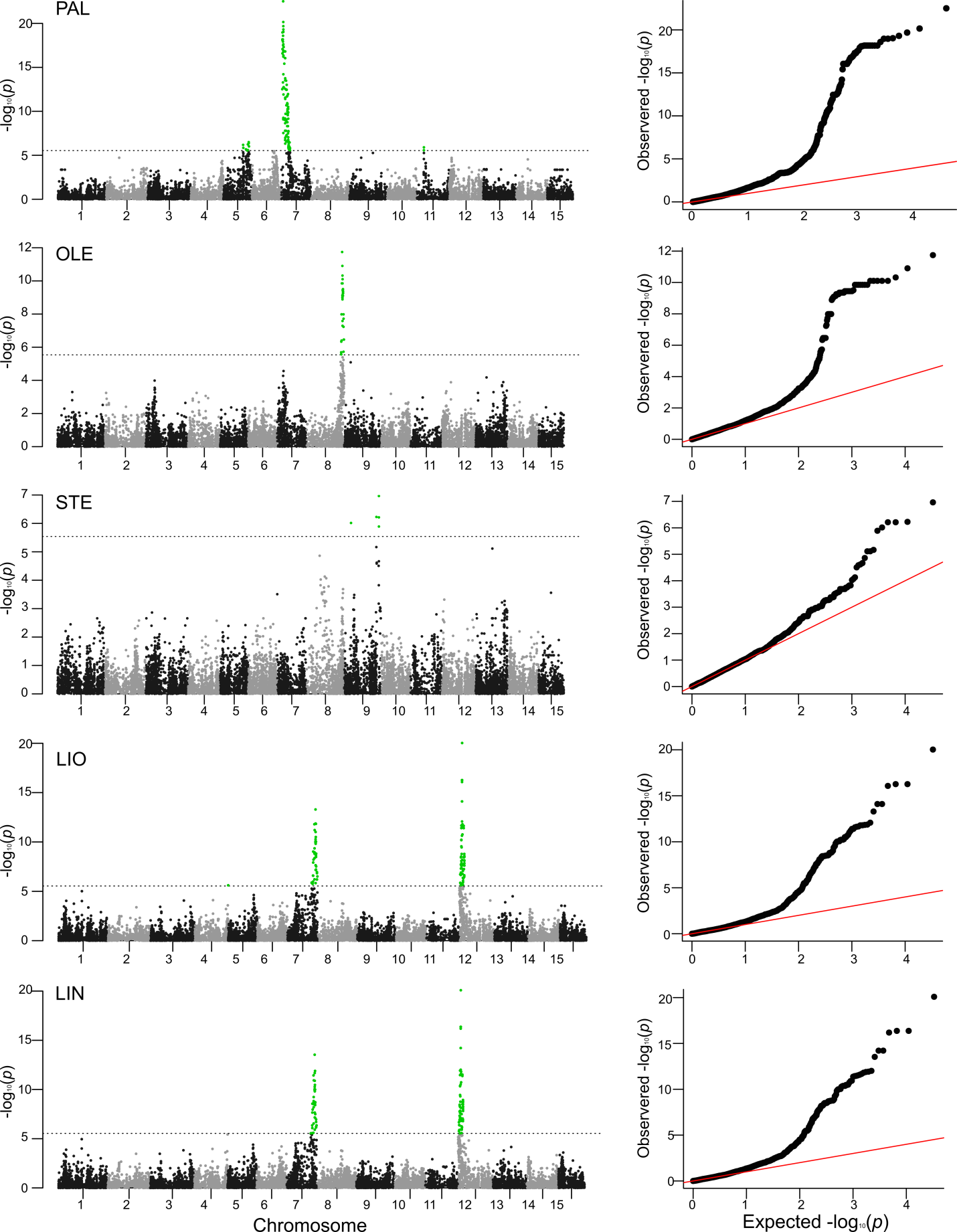


**Figure S1.** Manhattan and quantile-quantile (Q-Q) plots of 10 traits using BLUP values of traits estimated from six or eight environments in the BM+EV+SU population using GLM. PLH: plant height; DTM: days to maturity; PRO: protein content (%); OIL: oil content (%); IOD: iodine value; PAL: palmitic acid content (%); STE: stearic acid content (%); OLE: oleic acid content (%); LIO: linoleic acid content (%); LIN: linolenic acid content (%).


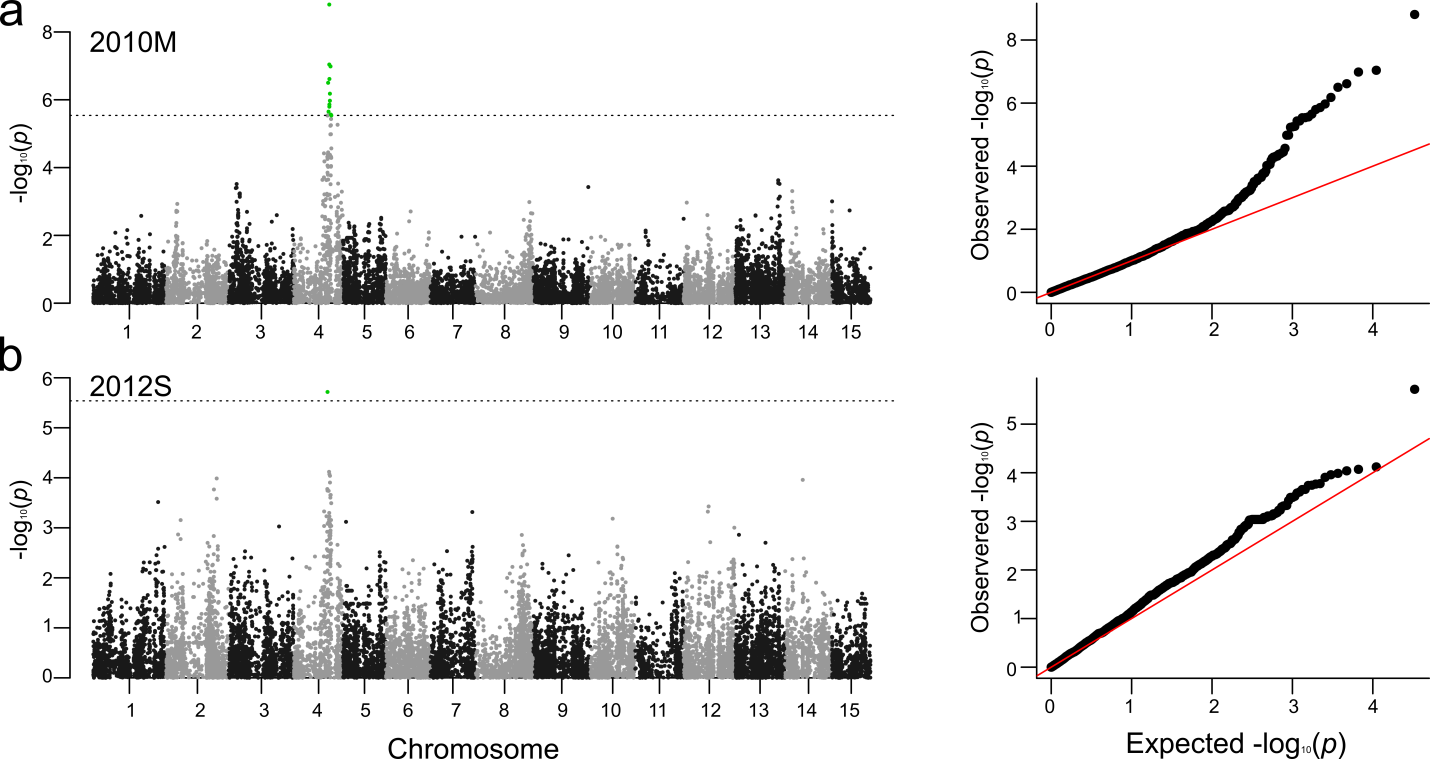
 **Figure S2.** Manhattan and quantile-quantile (Q-Q) plots of seed yield (YLD) using the BM+EV+SU combined population and GLM in two environments: (**a**) Morden 2010 and (**b**) Saskatoon 2012 showing the same QTL on chromosome 4.

**
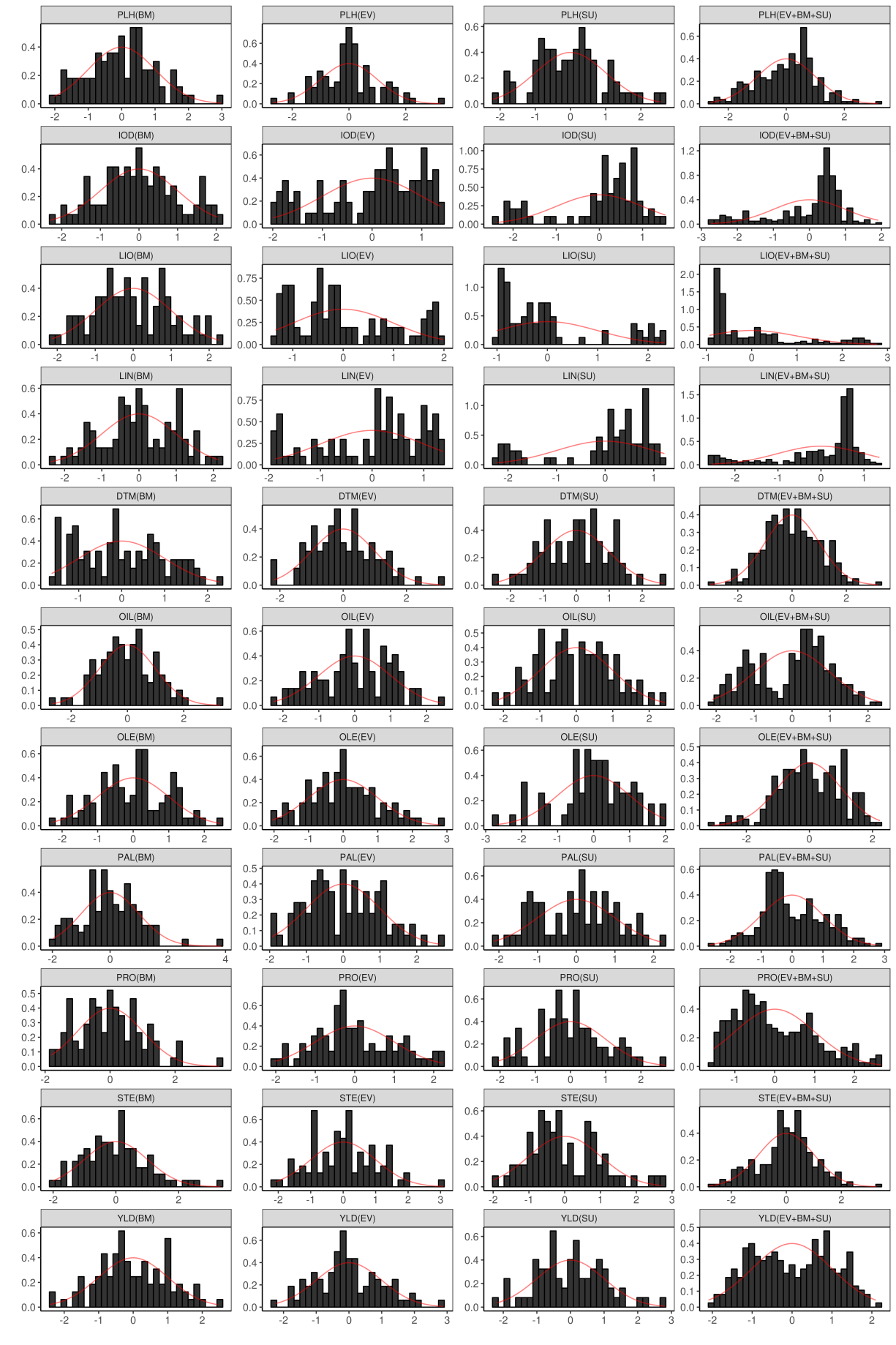
**

**Figure S3.** Histograms of 11 seed yield related and seed quality traits. A normal curve is fitted for each histogram.
