# Supplementary material for: Genome-Wide Association Study and Selection Signatures Detect Genomic Regions Associated with Seed Yield and Oil Quality in Flax
Source: Int J Mol Sci. 2018 Aug 6;19(8):2303. doi: 10.3390/ijms19082303 (PMC6121305; doi:10.3390/ijms19082303)
Supplement: Supplementary file 1 [file ijms-19-02303-s001.zip › ijms-338884-supplementary-final check/Supplementary_tables_8-final check.docx]

**Genome-wide association study and selection signatures detect genomic regions associated
with seed yield and oil quality in flax**

**Frank M. You ^1,2,^*, Jin Xiao ^1,3^, Pingchuan Li ^1^, Zhen Yao ^2^, Gaofeng Jia ^1,4^, Liqiang He ^1^,
Santosh Kumar ^5^, Braulio Soto-Cerda ^6,7^, Scott D. Duguid ^2^, Helen M. Booker ^4^, Khalid Y. Rashid ^2^
and Sylvie Cloutier ^1,6,^***

^1^ Ottawa Research and Development Centre, Agriculture and Agri-Food Canada, Ottawa, ON K1A 0C6, Canada; [xiaojin@njau.edu.cn](mailto:xiaojin@njau.edu.cn) (J.X.); [lipingchuan@gmail.com](mailto:lipingchuan@gmail.com) (P.L.); [gaofeng.jia@usask.ca](mailto:gaofeng.jia@usask.ca) (G.J.); [liqiang.he@canada.ca](mailto:liqiang.he@canada.ca) (L.H.)

^2^ Morden Research and Development Centre, Agriculture and Agri-Food Canada, Morden, MB R6M 1Y5, Canada; [zhen.yao@canada.ca](mailto:zhen.yao@canada.ca) (Z.Y.); [scott.duguid@agr.gc.ca](mailto:scott.duguid@agr.gc.ca) (S.D.D.); [khalid.rashid@agr.gc.ca](mailto:khalid.rashid@agr.gc.ca) (K.Y.R.)

^3^ Department of Agronomy, Nanjing Agricultural University, Nanjing 210095, China

^4^ Crop Development Centre, University of Saskatchewan, Saskatoon, SK S7N 5A8, Canada; helen.booker@usask.ca

^5^ Brandon Research and Development Centre, Agriculture and Agri-Food Canada, Brandon, MB R7A 5Y3, Canada; [Santosh.kumar@agr.gc.ca](mailto:Santosh.kumar@agr.gc.ca)

^6^ Department of Plant Science, University of Manitoba, Winnipeg, MB R3T 2N2, Canada; [braulio.soto@cgna.cl](mailto:braulio.soto@cgna.cl)

^7^ Agriaquaculture Nutritional Genomic Center, CGNA, Temuco 4871158, Chile

***** Correspondence: [frank.you@agr.gc.ca](mailto:frank.you@agr.gc.ca) (F.M.Y.); [sylvie.cloutuer@agr.gc.ca](mailto:sylvie.cloutuer@agr.gc.ca) (S.C.);
Tel.: +1-613-759-1539 (F.M.Y.); +1-613-759-1744 (S.C.)

**Supplementary Tables 1-5; 8-9; 13**

**Table S1.** Statistics of Illumina sequencing for 260 individuals from three bi-parental mapping populations.

|  | **Total** | **^2^**$\bar{\boldsymbol{x}}$ **± *s*** | **Range** |
| --- | --- | --- | --- |
| No. of reads (millions) | 15,109.4 | 57.7 ± 42.4 | 2.6–195.5 |
| Total read size (Mb) | 1,507,558.0 | 5754.0 ± 4250.1 | 262.3–19,545.2 |
| Genome coverage depth (×)^1^ | 4980.7 | 19.2 ± 14.1 | 0.87–64.7 |

^1^estimated based on the 302 Mb of scaffold sequences used as a reference for mapping the initial read; ^2^*s*: standard deviation.

**Table S2.** Distribution of SNPs identified across three bi-parental populations consisting of 260 individuals**.**

| **Chromosome** | **Genetic region** | | | **Total SNPs** | **SNP-associated genes** |
| --- | --- | --- | --- | --- | --- |
|  | **Exon** | **Intron** | **Intergenic** |  |  |
| 1 | 187 | 282 | 1043 | 1512 | 291 |
| 2 | 132 | 191 | 1210 | 1533 | 180 |
| 3 | 159 | 235 | 931 | 1325 | 239 |
| 4 | 124 | 156 | 544 | 824 | 156 |
| 5 | 147 | 188 | 737 | 1072 | 191 |
| 6 | 121 | 177 | 876 | 1174 | 180 |
| 7 | 115 | 138 | 593 | 846 | 162 |
| 8 | 206 | 170 | 714 | 1090 | 229 |
| 9 | 174 | 168 | 1069 | 1411 | 205 |
| 10 | 95 | 141 | 888 | 1124 | 141 |
| 11 | 111 | 108 | 382 | 601 | 156 |
| 12 | 168 | 236 | 981 | 1385 | 211 |
| 13 | 169 | 250 | 1153 | 1572 | 234 |
| 14 | 182 | 220 | 673 | 1075 | 229 |
| 15 | 111 | 137 | 496 | 744 | 152 |
| Total | 2201 | 2797 | 12,290 | 17,288 | 2956 |

**Table S3.** LD decay rate at the half of the maximum LD, average LD *r^2^* over entire genome and haplotype blocks for three bi-parental and the combined populations.

| **Population** | **LD decay rate**  **(kb)** | **Mean *r^2^* ± *sd*** | **Haplotype blocks** | | |
| --- | --- | --- | --- | --- | --- |
|  |  |  | **Total number of blocks** | **Mean block size (kb)** | **Max block size (kb)** |
| BM+EV+SU | 272 | 0.30 ± 0.25 | 1205 | 29.61 | 1161.72 |
| BM | 1242 | 0.37 ± 0.23 | 599 | 60.83 | 1853.89 |
| EV | 223 | 0.26 ± 0.24 | 648 | 35.45 | 1328.46 |
| SU | 728 | 0.28 ± 0.29 | 206 | 173.72 | 1761.73 |

**Table S4.** QTL identified from the three individual populations BM, EV and SU and from the combined population.

| Trait | Population/ Model | QTL No. | Chr | No of SNPs | Start position (bp) | End position (bp) | Peak SNP | Peak position (bp) | Peak allele1 | Peak allele2 | Peak effect | -log(*P*) | $h_{QTL}^{2}$ ± *s* |
| --- | --- | --- | --- | --- | --- | --- | --- | --- | --- | --- | --- | --- | --- |
| YLD  (t ha^-1^) | All/GLM^a^ | 1 | 4 | 11 | 13,831,239 | 14,968,389 | Lu4-14306304 | 14,306,304 | G | T | -4.91 | 8.80 | 0.14 ± 0.09 |
|  | All/GLM^b^ | 1 | 4 | 1 | 13,594,936 | 13,594,936 | Lu4-13594936 | 13,594,936 | C | T | -2.50 | 5.72 | 0.09 ± 0.12 |
| PLH  (cm) | EV/GLM | 2 | 1 | 4 | 13,887,715 | 13,930,292 | Lu1-13930280 | 13,930,280 | A | C | 9.32 | 6.31 | 0.21 ± 0.19 |
|  | BM/GLM | 3 | 1 | 1 | 20,012,490 | 20,012,490 | Lu1-20012490 | 20,012,490 | A | T | 4.51 | 5.58 | 0.20 ± 0.24 |
|  | BM/GLM | 4 | 4 | 11 | 14,305,982 | 15,042,104 | Lu4-14305982 | 14,305,982 | C | G | 4.29 | 7.21 | 0.12 ± 0.10 |
|  | All/GLM | 5 | 13 | 1 | 17,243,884 | 17,243,884 | Lu13-17243884 | 17,243,884 | T | G | -6.28 | 6.19 | 0.08 ± 0.11 |
|  | EV/GLM | 6 | 14 | 1 | 2,320,469 | 2,320,469 | Lu14-2320469 | 2,320,469 | C | G | -8.06 | 6.52 | 0.27 ± 0.29 |
| DTM  (days) | All/GLM | 7 | 4 | 5 | 13,171,757 | 14,809,179 | Lu4-13594936 | 13,594,936 | C | T | -2.16 | 6.38 | 0.10 ± 0.07 |
|  | BM/GLM | 7 | 4 | 12 | 13,512,423 | 15,042,104 | Lu4-14306304 | 14,306,304 | G | T | 2.73 | 10.84 | 0.18 ± 0.13 |
|  | All/MLM | 7 | 4 | 3 | 14,305,982 | 14,561,815 | Lu4-14306304 | 14,306,304 | G | T | 2.68 | 6.90 | 0.06 ± 0.06 |
|  | BM/MLM | 7 | 4 | 2 | 14,305,982 | 14,306,304 | Lu4-14306304 | 14,306,304 | G | T | 2.88 | 6.63 | 0.18 ± 0.19 |
|  | EV/GLM | 8 | 11 | 1 | 14,768,686 | 14,768,686 | Lu11-14768686 | 14,768,686 | T | C | -2.65 | 6.01 | 0.18 ± 0.22 |
| PRO  (%) | All/GLM | 9 | 15 | 2 | 14,746,288 | 14,746,310 | Lu15-14746310 | 14,746,310 | T | C | -0.97 | 6.49 | 0.12 ± 0.16 |
|  | EV/GLM | 9 | 15 | 1 | 14,746,310 | 14,746,310 | Lu15-14746310 | 14,746,310 | T | C | -1.05 | 5.56 | 0.09 ± 0.12 |
| OIL  (%) | All/GLM | 10 | 2 | 1 | 21,913,720 | 21,913,720 | Lu2-21913720 | 21,913,720 | G | A | -3.57 | 7.05 | 0.14 ± 0.19 |
|  | All/GLM | 11 | 5 | 3 | 15,704,607 | 15,705,039 | Lu5-15704628 | 15,704,628 | A | C | -2.57 | 8.49 | 0.34 ± 0.20 |
|  | All/MLM | 11 | 5 | 1 | 15,704,628 | 15,704,628 | Lu5-15704628 | 15,704,628 | A | C | -2.32 | 5.86 | 0.35 ± 0.33 |
|  | All/GLM | 12 | 6 | 1 | 4,879,632 | 4,879,632 | Lu6-4879632 | 4,879,632 | A | G | 4.27 | 6.40 | 0.12 ± 0.17 |
|  | All/GLM | 13 | 6 | 5 | 13,799,180 | 13,970,951 | Lu6-13970520 | 13,970,520 | A | T | 4.29 | 6.82 | 0.17 ± 0.20 |
|  | All/GLM | 14 | 7 | 1 | 14,209,179 | 14,209,179 | Lu7-14209179 | 14,209,179 | G | A | -5.88 | 6.53 | 0.45 ± 0.37 |
|  | All/GLM | 15 | 10 | 1 | 6,517,448 | 6,517,448 | Lu10-6517448 | 6,517,448 | T | C | -2.72 | 5.66 | 0.39 ± 0.35 |
|  | All/GLM | 16 | 12 | 28 | 4,591,214 | 7,491,405 | Lu12-6708984 | 6,708,984 | G | A | -5.91 | 6.42 | 0.04 ± 0.07 |
|  | EV/GLM | 17 | 15 | 4 | 14,665,900 | 15,429,055 | Lu15-14665900 | 14,665,900 | T | C | -1.14 | 7.71 | 0.08 ± 0.08 |
| IOD | All/MLM | 18 | 4 | 1 | 19,909,467 | 19,909,467 | Lu4-19909467 | 19,909,467 | G | A | -26.80 | 5.58 | 0.42 ± 0.36 |
|  | All/GLM | 19 | 7 | 40 | 15,346,458 | 17,977,459 | Lu7-16848770 | 16,848,770 | A | C | 16.10 | 13.51 | 0.54 ± 0.13 |
|  | SU/GLM | 19 | 7 | 27 | 15,425,129 | 17,226,132 | Lu7-16817334 | 16,817,334 | A | G | -19.31 | 7.18 | 0.12 ± 0.12 |
|  | EV/GLM | 19 | 7 | 3 | 15,922,671 | 16,233,470 | Lu7-16233470 | 16,233,470 | G | T | -18.98 | 6.49 | 0.11 ± 0.11 |
|  | All/MLM | 19 | 7 | 5 | 16,158,586 | 17,083,123 | Lu7-16848770 | 16,848,770 | A | C | 14.34 | 7.71 | 0.28 ± 0.15 |
|  | All/GLM | 20 | 12 | 72 | 489,561 | 2,981,642 | Lu12-1633507 | 1,633,507 | G | A | -15.60 | 19.62 | 0.29 ± 0.11 |
|  | All/MLM | 20 | 12 | 35 | 1,172,436 | 2,981,642 | Lu12-1542711 | 1,542,711 | A | G | 18.85 | 10.51 | 0.30 ± 0.14 |
|  | EV/GLM | 20 | 12 | 56 | 1,172,436 | 2,981,642 | Lu12-1633507 | 1,633,507 | G | A | -17.88 | 9.47 | 0.15 ± 0.10 |
|  | SU/GLM | 20 | 12 | 3 | 1,267,375 | 1,633,507 | Lu12-1633507 | 1,633,507 | G | A | -16.90 | 5.59 | 0.14 ± 0.19 |
| PAL  (%) | All/GLM | 21 | 5 | 2 | 12,062,376 | 12,182,441 | Lu5-12062376 | 12,062,376 | G | A | -0.36 | 6.18 | 0.08 ± 0.09 |
|  | All/GLM | 22 | 5 | 7 | 13,797,851 | 15,668,995 | Lu5-15503464 | 15,503,464 | C | G | -0.41 | 6.49 | 0.10 ± 0.08 |
|  | All/GLM | 23 | 7 | 123 | 624,461 | 5,423,691 | Lu7-1187005 | 1,187,005 | A | G | 0.58 | 22.51 | 0.39 ± 0.12 |
|  | SU/GLM | 23 | 7 | 84 | 624,461 | 4,186,137 | Lu7-747411 | 747,411 | T | C | -0.73 | 10.73 | 0.56 ± 0.18 |
|  | All/MLM | 23 | 7 | 10 | 747,411 | 1,320,171 | Lu7-1187005 | 1,187,005 | A | G | 0.40 | 8.09 | 0.20 ± 0.12 |
|  | EV/GLM | 23 | 7 | 3 | 969,909 | 970,231 | Lu7-969909 | 969,909 | T | C | -0.35 | 6.89 | 0.09 ± 0.11 |
|  | EV/MLM | 23 | 7 | 3 | 969,909 | 970,231 | Lu7-969932 | 969,932 | T | C | -0.37 | 5.87 | 0.09 ± 0.11 |
|  | All/GLM | 24 | 11 | 2 | 4,417,685 | 4,429,424 | Lu11-4429424 | 4,429,424 | A | T | 0.40 | 5.90 | 0.07 ± 0.09 |
| OLE  (%) | All/GLM | 25 | 8 | 65 | 21,782,841 | 23,527,563 | Lu8-22386322 | 22,386,322 | A | G | 2.45 | 11.74 | 0.16 ± 0.13 |
|  | SU/GLM | 25 | 8 | 104 | 21,819,178 | 23,298,218 | Lu8-22386322 | 22,386,322 | A | G | 2.42 | 8.37 | 0.20 ± 0.19 |
| STE  (%) | All/GLM | 26 | 9 | 1 | 4,229,230 | 4,229,230 | Lu9-4229230 | 4,229,230 | C | A | -1.22 | 6.01 | 0.51 ± 0.37 |
|  | All/GLM | 27 | 9 | 5 | 20,080,531 | 21,636,823 | Lu9-21627283 | 21,627,283 | A | G | 0.39 | 6.96 | 0.08 ± 0.07 |
|  | All/MLM | 27 | 9 | 2 | 20,080,531 | 21,627,283 | Lu9-21627283 | 21,627,283 | A | G | 0.42 | 6.25 | 0.10 ± 0.10 |
| LIO  (%) | All/GLM | 28 | 4 | 1 | 19,909,467 | 19,909,467 | Lu4-19909467 | 19,909,467 | A | G | 33.05 | 5.61 | 0.45 ± 0.36 |
|  | All/GLM | 29 | 7 | 45 | 14,540,706 | 17,977,459 | Lu7-16848770 | 16,848,770 | A | C | -17.90 | 13.29 | 0.66 ± 0.11 |
|  | SU/GLM | 29 | 7 | 29 | 15,425,129 | 17,226,132 | Lu7-16817334 | 16,817,334 | A | G | 22.18 | 7.12 | 0.12 ± 0.12 |
|  | EV/GLM | 29 | 7 | 3 | 15,922,671 | 16,233,470 | Lu7-16233470 | 16,233,470 | G | T | -20.10 | 5.96 | 0.11 ± 0.11 |
|  | All/MLM | 29 | 7 | 3 | 16,222,925 | 16,887,060 | Lu7-16848770 | 16,848,770 | A | C | -14.00 | 6.10 | 0.37 ± 0.21 |
|  | All/GLM | 30 | 12 | 75 | 489,561 | 2,981,642 | Lu12-1633507 | 1,633,507 | G | A | -17.75 | 20.03 | 0.29 ± 0.11 |
|  | All/MLM | 30 | 12 | 61 | 1,172,436 | 2,981,642 | Lu12-1542711 | 1,542,711 | A | G | -23.50 | 15.00 | 0.34 ± 0.12 |
|  | EV/GLM | 30 | 12 | 60 | 1,172,436 | 2,981,642 | Lu12-1176253 | 1,176,253 | C | T | -20.35 | 9.88 | 0.17 ± 0.11 |
|  | SU/GLM | 30 | 12 | 3 | 1,267,375 | 1,633,507 | Lu12-1633507 | 1,633,507 | G | A | -19.50 | 5.60 | 0.14 ± 0.19 |
|  | EV/MLM | 30 | 12 | 1 | 2,579,513 | 2,579,513 | Lu12-2579513 | 2,579,513 | C | T | -18.64 | 5.54 | 0.13 ± 0.16 |
| LIN  (%) | All/MLM | 31 | 4 | 1 | 19,909,467 | 19,909,467 | Lu4-19909467 | 19,909,467 | A | G | -30.70 | 5.91 | 0.44 ± 0.36 |
|  | All/GLM | 32 | 7 | 43 | 14,540,719 | 17,977,459 | Lu7-16848770 | 16,848,770 | A | C | 17.90 | 13.53 | 0.52 ± 0.13 |
|  | SU/GLM | 32 | 7 | 29 | 15,425,129 | 17,226,132 | Lu7-16817334 | 16,817,334 | A | G | -21.89 | 7.22 | 0.12 ± 0.12 |
|  | EV/GLM | 32 | 7 | 3 | 15,922,671 | 16,233,470 | Lu7-16233470 | 16,233,470 | G | T | 20.50 | 6.18 | 0.11 ± 0.11 |
|  | All/MLM | 32 | 7 | 5 | 16,158,586 | 17,083,123 | Lu7-16848770 | 16,848,770 | A | C | 15.87 | 7.62 | 0.29 ± 0.16 |
|  | All/GLM | 33 | 12 | 75 | 489,561 | 2,981,642 | Lu12-1633507 | 1,633,507 | G | A | -17.60 | 20.09 | 0.28 ± 0.11 |
|  | All/MLM | 33 | 12 | 35 | 1,172,436 | 2,981,642 | Lu12-1574479 | 1,574,479 | G | T | 20.87 | 10.73 | 0.35 ± 0.15 |
|  | EV/GLM | 33 | 12 | 60 | 1,172,436 | 2,981,642 | Lu12-1633507 | 1,633,507 | G | A | -19.80 | 9.59 | 0.17 ± 0.11 |
|  | SU/GLM | 33 | 12 | 3 | 1,267,375 | 1,633,507 | Lu12-1633507 | 1,633,507 | G | A | -19.24 | 5.67 | 0.15 ± 0.19 |

Abbreviations for traits are listed in Table 1. BM: a recombinant inbred line (RIL) population of 96 F_2:6_ lines from the cross CDC Bethune/Macbeth; EV: an RIL population of 96 F_2:6_ lines from the cross E1747/ Viking; SU: a doubled haploid (DH) population of 90 lines from the cross SP2047/ UGG5-5; All: the combined individuals of the three populations, BM+EV+SU. For YLD, QTL were identified only using the phenotyping data obtained in Morden in 2010 (a) and Saskatoon in 2012 (b). For all other traits, BLUP values from six to eight environments were used. No.: number.

**Table S5.** Number of QTL identified from different populations or statistical models.

|  | **Trait** | | | | | | | | | | | **Total** |
| --- | --- | --- | --- | --- | --- | --- | --- | --- | --- | --- | --- | --- |
|  | **YLD** | **PLH** | **DTM** | **PRO** | **OIL** | **IOD** | **PAL** | **OLE** | **STE** | **LIO** | **LIN** |  |
| Population |  |  |  |  |  |  |  |  |  |  |  |  |
| BM+EV+SU | 1 | 1 | 1 | 1 | 7 | 3 | 4 | 1 | 2 | 3 | 3 | 27 |
| BM |  | 2 | 1 |  |  |  |  |  |  |  |  | 3 |
| EV |  | 2 | 1 | 1 | 1 | 2 | 1 |  |  | 2 | 2 | 12 |
| SU |  |  |  |  |  | 2 | 1 | 1 |  | 2 | 2 | 8 |
| Total (non-redundant) | 1 | 5 | 2 | 1 | 8 | 3 | 4 | 1 | 2 | 3 | 3 | 33 |
| Statistical model |  |  |  |  |  |  |  |  |  |  |  |  |
| GLM | 1 | 5 | 2 | 1 | 8 | 2 | 4 | 1 | 2 | 3 | 2 | 31 |
| MLM |  |  | 1 |  | 1 | 3 | 1 |  | 1 | 3 | 3 | 13 |
| Total (non-redundant) | 1 | 5 | 2 | 1 | 8 | 3 | 4 | 1 | 2 | 3 | 3 | 33 |

**Table S8.** Pleiotropic or linkage relationship of QTL between several traits.

| Locus | Trait | QTL No. | QTL name | Chr | Start position | End position | Peak SNP | Peak position | $h_{QTL}^{2}$ | Peak allele1 | Peak allele2 |
| --- | --- | --- | --- | --- | --- | --- | --- | --- | --- | --- | --- |
| 1 | PLH | 4 | *QPLH-Lu4.3* | 4 | 14,305,982 | 15,042,104 | Lu4-14305982 | 14,305,982 | 0.12 | C | G |
|  | DTM | 7 | *QDTM-Lu4.1* | 4 | 14,305,982 | 14,561,815 | Lu4-14306304 | 14,306,304 | 0.06 | G | T |
|  | YLD | 1 | *QYLD-Lu4.1* | 4 | 13,831,239 | 14,968,389 | Lu4-14306304 | 14,306,304 | 0.14 | G | T |
| 2 | IOD | 18 | *QIOD-Lu4.1* | 4 | 19,909,467 | 19,909,467 | Lu4-19909467 | 19,909,467 | 0.42 | A | G |
|  | LIO | 28 | *QLIO-Lu4.1* | 4 | 19,909,467 | 19,909,467 | Lu4-19909467 | 19,909,467 | 0.45 | A | G |
|  | LIN | 31 | *QLIN-Lu4.1* | 4 | 19,909,467 | 19,909,467 | Lu4-19909467 | 19,909,467 | 0.44 | A | G |
| 3 | IOD | 19 | *QIOD-Lu7.2* | 7 | 15,346,458 | 17,977,459 | Lu7-16848770 | 16,848,770 | 0.54 | A | C |
|  | LIO | 29 | *QLIO-Lu7.2* | 7 | 14,540,706 | 17,977,459 | Lu7-16848770 | 16,848,770 | 0.66 | A | C |
|  | LIN | 32 | *QLIN-Lu7.2* | 7 | 14,540,719 | 17,977,459 | Lu7-16848770 | 16,848,770 | 0.52 | A | C |
| 4 | IOD | 20 | *QIOD-Lu12.3* | 12 | 489,561 | 2,981,642 | Lu12-1633507 | 1,633,507 | 0.29 | A | G |
|  | LIO | 30 | *QLIO-Lu12.3* | 12 | 489,561 | 2,981,642 | Lu12-1633507 | 1,633,507 | 0.29 | A | G |
|  | LIN | 33 | *QLIN-Lu12.3* | 12 | 489,561 | 2,981,642 | Lu12-1633507 | 1,633,507 | 0.28 | A | G |
| 5 | PRO | 9 | *QPRO-Lu15.1* | 15 | 14,746,310 | 14,746,310 | Lu15-14746310 | 14,746,310 | 0.12 | C | T |
|  | OIL | 17 | *QOIL-Lu15.7* | 15 | 14,665,900 | 15,429,055 | Lu15-14665900 | 14,665,900 | 0.08 | C | T |
| 6 | PAL | 22 | *QPAL-Lu5.2* | 5 | 13,797,851 | 15,668,995 | Lu5-15503464 | 15,503,464 | 0.10 | C | G |
|  | OIL | 11 | *QOIL-Lu5.2* | 5 | 15,704,607 | 15,705,039 | Lu5-15704628 | 15,704,628 | 0.34 | A | C |

**Table S9.** Comparison of 11 traits for their population means and standard deviations ($\bar{x}$ ± *s*) among three mapping populations.

| Trait | Population | | |
| --- | --- | --- | --- |
|  | BM | EV | SU |
| YLD (t ha^-1^) | 15.81 ± 1.14 a | 10.82 ± 1.26 c | 13.03 ± 1.70 b |
| PLH (cm) | 58.28 ± 3.57 b | 60.36 ± 4.58 a | 50.04 ± 3.84 c |
| DTM (days) | 95.93 ± 2.00 a | 93.81 ± 1.98 c | 94.81 ± 3.05 b |
| PRO (%) | 24.48 ± 0.58 c | 27.20 ± 1.03 a | 25.31 ± 0.88 b |
| OIL (%) | 45.06 ± 0.88 b | 41.25 ± 0.94 c | 46.32 ± 1.33 a |
| IOD | 190.38 ± 2.40 a | 173.07 ± 14.69 c | 186.06 ± 16.19 b |
| PAL (%) | 4.81 ± 0.13 c | 5.54 ± 0.33 a | 4.91 ± 0.53 b |
| OLE (%) | 19.92 ± 1.16 a | 17.33 ± 1.07 b | 15.73 ± 1.65 c |
| STE (%) | 3.72 ± 0.27 b | 3.90 ± 0.50 a | 3.01 ± 0.51 c |
| LIO (%) | 15.73 ± 0.68 c | 37.78 ± 16.23 a | 30.78 ± 18.67 b |
| LIN (%) | 55.82 ± 1.42 a | 35.44 ± 16.26 b | 45.58 ± 18.31 c |

The different letters represent statistical significance among three populations at 0.01 probability level.

**Table S13.** Simple correlations among 11 traits using BLUP values over eight environments of the merged population.

|  | PLH | DTM | PRO | OIL | IOD | PAL | STE | OLE | LIO | LIN |
| --- | --- | --- | --- | --- | --- | --- | --- | --- | --- | --- |
| YLD | -0.09 | 0.23** | -0.73** | 0.57** | 0.46** | -0.57** | -0.09 | 0.50** | -0.51** | 0.49** |
| PLH |  | 0.30** | 0.20** | -0.54** | -0.15* | 0.27** | 0.55** | 0.40** | -0.04 | -0.04 |
| DTM |  |  | -0.41** | 0.24** | 0.10 | -0.08 | 0.19** | 0.31** | -0.19** | 0.15* |
| PRO |  |  |  | -0.70** | -0.53** | 0.54** | 0.20** | -0.34** | 0.53** | -0.53** |
| OIL |  |  |  |  | 0.37** | -0.57** | -0.45** | 0.04 | -0.27** | 0.31** |
| IOD |  |  |  |  |  | -0.63** | -0.23** | 0.22** | -0.95** | 0.98** |
| PAL |  |  |  |  |  |  | 0.24** | -0.29** | 0.57** | -0.59** |
| STE |  |  |  |  |  |  |  | 0.42** | 0.00 | -0.10 |
| OLE |  |  |  |  |  |  |  |  | -0.49** | 0.37** |
| LIO |  |  |  |  |  |  |  |  |  | -0.99** |

* and ** represent the significance at 0.05 and 0.01 probability level, respectively. The sample size is 258.
